# Neurodevelopmental trajectories of letter and speech sound processing from preschool to the end of elementary school

**DOI:** 10.1016/j.dcn.2023.101255

**Published:** 2023-05-12

**Authors:** S.V. Di Pietro, I.I. Karipidis, G. Pleisch, S. Brem

**Affiliations:** a Department of Child and Adolescent Psychiatry and Psychotherapy, University Hospital of Psychiatry Zurich, University of Zurich, Switzerland; b Neuroscience Center Zurich, University of Zurich and ETH Zurich, Switzerland; c URPP Adaptive Brain Circuits in Development and Learning (AdaBD), University of Zurich, Zurich, Switzerland

**Keywords:** Audiovisual integration, Superior temporal gyrus, Visual word form area, FMRI

## Abstract

Learning to read alphabetic languages starts with learning letter–speech-sound associations. How this process changes brain function during development is still largely unknown. We followed 102 children with varying reading skills in a mixed-longitudinal/cross-sectional design from the prereading stage to the end of elementary school over five time points (n = 46 with two and more time points, of which n = 16 fully-longitudinal) to investigate the neural trajectories of letter and speech sound processing using fMRI. Children were presented with letters and speech sounds visually, auditorily, and audiovisually in kindergarten (6.7yo), at the middle (7.3yo) and end of first grade (7.6yo), and in second (8.4yo) and fifth grades (11.5yo). Activation of the ventral occipitotemporal cortex for visual and audiovisual processing followed a complex trajectory, with two peaks in first and fifth grades. The superior temporal gyrus (STG) showed an inverted U-shaped trajectory for audiovisual letter processing, a development that in poor readers was attenuated in middle STG and absent in posterior STG. Finally, the trajectories for letter-speech-sound integration were modulated by reading skills and showed differing directionality in the congruency effect depending on the time point. This unprecedented study captures the development of letter processing across elementary school and its neural trajectories in children with varying reading skills.

## Highlights

• Neurodevelopmental trajectories of audiovisual (AV) letter processing.
• Measurements at five time points from preschool across primary school with fMRI.
• Activation in ventral occipitotemporal cortex followed a complex trajectory.
• Activation in superior temporal gyrus followed an inverted U-shaped development.
• Trajectories for AV processing and integration were modulated by reading skills.

## 1 Introduction

Letters form the basis of scripts in alphabetic languages, and learning the associations between letters and speech sounds is therefore essential for successful reading acquisition. Children’s language development involves their early exposure to speech sounds as parts of words long before formal reading instruction. In primary school, the familiar speech sounds are matched to less familiar letters during reading acquisition (Blomert, 2011). This process is the first step in formal reading instruction and provides a foundation for letter-by-letter processing, which with increasing reading experience enables fast automatic word recognition (Houdé et al., 2010, McCandliss et al., 2003, Snowling and Hulme, 2005, Ziegler and Goswami, 2005).

During reading acquisition, the language and reading networks of the brain undergo functional reorganization and specialization (Martin et al., 2015): Changes in brain activation of and connectivity between visual and auditory language processing regions emerge as soon as letter–speech-sound associations are learnt (Blau et al., 2010, Brem et al., 2010, Chyl et al., 2018, Karipidis et al., 2017). However, full automatization of letter–speech-sound associations and the corresponding maturation of the underlying brain systems is a lengthy process that takes several years (Dehaene et al., 2015, Froyen et al., 2009). To date, few studies have investigated and characterized changes in functional brain activation during the development of single-letter and speech-sound processing and in parallel with reading acquisition (Blackburne et al., 2014, Caffarra et al., 2021, Cantlon et al., 2011, Centanni et al., 2018, Fraga-González et al., 2021, Froyen et al., 2009, Karipidis et al., 2018, Karipidis et al., 2021, Pleisch et al., 2019, Plewko et al., 2018, Romanovska et al., 2022, Zaric et al., 2015).

Previous research has proposed a predominantly left-lateralized reading network for reading and letter–speech-sound processing and integration within the brain, consisting of visual and auditory language areas and the attention system (Black et al., 2017, Price, 2012, Richlan, 2012, Richlan, 2019, van Atteveldt et al., 2004). Central to reading in the visual domain is the visual word form area (VWFA, Cohen et al., 2000), an area within the ventral occipitotemporal cortex (vOTC) that is specialized to process words. It shows preferential activation to print and whole word forms compared to control stimuli such as symbols and checkerboard patterns, and its importance for the reading process is widely accepted (Caffarra et al., 2021, Centanni et al., 2017, Cohen et al., 2000, Dehaene-Lambertz et al., 2018, Dehaene and Cohen, 2011, Lerma-Usabiaga et al., 2018, McCandliss et al., 2003, Turkeltaub et al., 2003, Vinckier et al., 2007). Previous studies have presented evidence indicating a posterior-to-anterior gradient in vOTC activation in which posterior regions are predominantly implicated in perceptual print and letter processing and anterior regions in lexical aspects of print processing (Brem et al., 2006, Caffarra et al., 2021, Lerma-Usabiaga et al., 2018, Taylor et al., 2019, Thesen et al., 2012, Vinckier et al., 2007, White et al., 2019). Visual specialization to written language emerges rapidly when children learn how to read (Brem et al., 2010, Chyl et al., 2019, Dehaene-Lambertz et al., 2018, Saygin et al., 2016) and continues to develop throughout childhood and adolescence. This development is associated with an enlargement of the activated volume of the respective parts of vOTC (Ben-Shachar et al., 2011, Nordt et al., 2021). Development of visual specialization in this area has also been reported to depend on reading skills (Brem et al., 2020, Dehaene et al., 2015, Richlan et al., 2009, Romanovska et al., 2021).

Whereas the vOTC is crucial for processing written language, the brain areas for processing speech and for audiovisual integration are mainly located within the superior temporal cortex, its sulcus and gyrus, and the primary auditory cortex (Beauchamp et al., 2004, Booth et al., 2001, Raij et al., 2000, van Atteveldt et al., 2004). When forming letter–speech-sound associations, superior temporal cortical (STC) regions form connections to higher-order visual regions, which then enable responses to print in addition to speech sounds (Bonte et al., 2017). Both the STC and vOTC, as well as other regions of the reading network such as the inferior frontal gyrus (IFG) and parietal regions, are thus tightly coupled and interact to process and integrate information efficiently through bottom-up and top-down feedback mechanisms. Previous studies have shown that functional and effective connectivity between areas of the reading network depends on the reading stage and reading skills (Bitan et al., 2009, Chen et al., 2019, Di Pietro et al., 2023, Morken et al., 2017, Wang et al., 2020, Wise Younger et al., 2017, Yu et al., 2018) and that aberrant connectivity within the reading network is linked to reading difficulties (Richlan, 2019).

During audiovisual processing, congruent pairs of letters and speech sounds have been shown to activate the posterior superior temporal gyrus (STG) and sulcus more than incongruent letter–speech-sound pairs (Raij et al., 2000, van Atteveldt et al., 2004). The response differences to congruent and incongruent audiovisual pairs, referred to as the congruency effect, have been associated with individual differences in reading skills and phonological awareness (Blau et al., 2010, Blau et al., 2009, Karipidis et al., 2018, Kronschnabel et al., 2014, McNorgan et al., 2014, McNorgan et al., 2013, Wang et al., 2020, Ye et al., 2017). Although the STG has been shown to contribute to audiovisual processing and integration, a process that is crucial to reading acquisition (Blomert, 2011, Caffarra et al., 2021, Holloway et al., 2015, Plewko et al., 2018), little is known about the longitudinal, developmental changes in audiovisual integration and the brain circuitry involved in increasing reading practice and expertise.

Visual specialization to print has been proposed to follow an inverted U-shaped development in typical reading acquisition, with an increase in activation in vOTC at the beginning of formal reading instruction, followed by a decrease with growing expertise; such inverted U-shaped developmental trajectories of neural activation may be explained by the predictive model account of vOTC function (Price and Devlin, 2011) and by the expansion-renormalization model of neural changes during skill acquisition (Lövdén et al., 2020). According to the predictive model account, expertise-dependent changes in prediction error signaling may account for the characteristic learning curve (Price and Devlin, 2011). The expansion-renormalization model explains such skill-learning trajectories as resulting from plastic changes occurring at different levels in the brain circuits involved in the process of learning, which progress from expansion and exploration to selection and refinement. An inverted U-shaped developmental trajectory of activation during reading acquisition has previously been observed in the visual N1 event-related potential (ERP) during word and letter processing measured with electroencephalography (EEG, Fraga-González et al., 2021; Maurer et al., 2006). Amplitudes in the occipitotemporal N1, the electrophysiological correlate of VWFA activity, peak at the beginning of primary school, in first grade for letters and second grade for words (Fraga-González et al., 2021, Maurer et al., 2006), when letter–speech-sound mapping is practiced intensively (Chyl et al., 2021). The inverted U-shaped development of VWFA activation to print has also been observed with functional magnetic resonance imaging (fMRI) measurements (Dehaene-Lambertz et al., 2018). Similarly, the left STG and left lateral inferior precentral gyrus show an inverted U-shaped development during audiovisual processing in 8–11-year-old children (Romanovska et al., 2022).

Despite an increasing number of studies investigating the development of print and language processing, longitudinal studies covering several time points over the course of primary school are still scarce (Chyl et al., 2021). Here, we are interested in the developmental trajectories of activation in brain regions involved in or contributing to letter processing over several stages of reading acquisition: At the prereading stage in children attending kindergarten, when letters are still unfamiliar, at the beginning of formal reading instruction, when letter-by-letter decoding is the main reading strategy, and at the end of formal reading instruction, when reading is automatized and sight word reading prevails. Using fMRI recordings collected during an implicit target detection task and by applying a combined longitudinal and cross-sectional analyses approach, we compared brain activation to visual, auditory, and audiovisual stimuli over five time points in kindergarten, first grade (two time points), second grade, and fifth grade of primary school in children with varying reading skills. From previous studies (Fraga-González et al., 2021, Karipidis et al., 2021, Maurer et al., 2006, Romanovska et al., 2022) and current models of skill learning and specialization (Lövdén et al., 2020, Price and Devlin, 2011, Wenger et al., 2017), we expected inverted U-shaped developments for the activation of auditory, visual, and audiovisual letter processing within temporal and vOTC regions of the reading network, with an activation increase from kindergarten, before the start of formal reading instruction, to first grade when children start to learn letter–speech-sound correspondences, and a decline in activation after initial consolidation of correspondences up to fifth grade. Given that inverted U-shaped trajectories might represent a change from effortful letter-by-letter processing to automatized reading, we presume that these developmental trajectories are modulated by children’s reading skills.

## 2 Methods

### 2.1 Participants

A sample of 105 children participated in this study at least on one of the following five time points: kindergarten (T1, 6.65 yo), middle of first grade (T2, 7.35 yo), end of first grade (T3, 7.64 yo), second grade (T4, 8.42 yo), and fifth grade (T5, 11.40 yo). Children in kindergarten (T1) did not receive any formal reading instruction yet and were therefore at prereading level. The data of three participants were completely excluded due to poor MRI data quality or missing behavioral data at all available time points. The remaining 102 participants formed the mixed longitudinal and cross-sectional group for our analyses (n_T1_=35, n_T2_=44, n_T3_=43, n_T4_=38, n_T5_=78). For these analyses we used all available datasets for which the fMRI data quality conformed with our strict data quality criteria as explained below (chapter 2.4): n = 15 * 5 time points, n = 16 * 4 time points, n = 13 * 3 time points, n = 2 * 2 time points, n = 56 * 1 time point, summing up to a total of 238 datasets. From the longitudinal/cross-sectional sample of 102 children who participated in at least one out of five time points, we had 24 children that participated in the fMRI and behavioral sessions at every time point (i.e. from T1 to T5; 78 children were missing one time point due to later enrolment in the study or discontinuation). From these 24 children, 5 had to be excluded due to artifacts from dental braces and 3 due to not meeting the fMRI motion quality criteria described below, leaving a final sample of 16 participants as the fully longitudinal group (9 female; for more detailed information about the demographics, see Table 1). Note, for the longitudinal analyses we applied a slightly more lenient motion criterion as compared with the mixed longitudinal and cross-sectional analyses, which allowed us to include the data of one more participant (see chapter 2.4).

**Table 1:** Demographic characteristics of study participants.

mixed longitudinal and cross-sectional sample (n = 102)					
	T1	T2	T3	T4	T5
n	35	44	43	38	78
Sex (female), No.	16 (46%)	23 (52%)	23 (53%)	20 (53%)	47 (60%)
Hand (right), No.	32 (91%)	40 (91%)	38 (88%)	33 (87%)	70 (90%)
Age in years	6.65 (.30), 6.13–7.30	7.35 (.32), 6.80–8.21	7.64 (.30), 7.08–8.47	8.42 (.32), 7.76–9.19	11.40 (.43), 9.99–12.16
Reading fluency raw values^1^		10.13 (9.30), 0.00–57.00	16.51 (10.06), 2.00–61.00	27.00 (11.69), 9.00–72.50	55.31 (20.45), 21.00–104.05
Reading fluency percentile^2^		27.30 (26.93), 0.50–99.00^a^	49.31 (25.90), 2.25–98.25	32.76 (22.73), 1.50–97.50	38.13 (32.16), 1.00-.96.50
Nonverbal IQ^3^	103.51 (7.21)^b^				

**longitudinal sample (n = 16)**					
Sex (female), No.	9 (56%)				
Hand (right), No.	15 (94%)				
Age in years	6.67 (.30), 6.13–7.17	7.31 (.30), 6.79–7.87	7.59 (.29), 7.08–8.11	8.41 (.29), 7.75–8.79	11.47 (.24), 10.89–11.81
Reading fluency raw values^1^		11.50 (12.90), 0.50–57.00	17.00 (13.10), 4.00–61.00	26.69 (14.61), 8.00–72.50	50.00 (17.64), 26.00–86.50
Reading fluency percentile^2^		24.97 (25.93), 0.50–99.00^a^	47.80 (24.74), 9.75–98.25	30.77 (25.84), 1.25–97.50	27.80 (28.60), 1.50–93.00
Nonverbal IQ^3^	102.38 (6.64)				

*Notes*. Data are presented as "mean (SD), range" if not indicated otherwise. Reading fluency raw values^1^ and percentile scores^2^ of average word and pseudoword reading (number of correctly read items per minute) measured with Salzburger Lese- und Rechtschreibtest II. Nonverbal IQ^3^ measured with Reynolds Intellectual Assessment Scales or the CFT1-R. ^a^percentile scores for T2 based on in house norms. ^b^value missing for one subject.

All participants had nonverbal IQ scores > 80 as measured with the Reynolds Intellectual Assessment Scales or the Culture Fair Intelligence Test (CFT1-R, Reynolds and Kamphaus, 2003; Weiß and Osterland, 2013, see chapter 2.2), normal or corrected to normal vision and did not show any neurological or psychiatric impairment, except for attention deficit hyperactivity disorder/attention deficit disorder (ADHD/ADD, n = 6 for the mixed longitudinal and cross-sectional sample, n = 2 for the longitudinal sample). Subjects with ADHD/ADD were either unmedicated or were asked to discontinue medical treatment at least 24 h before the measurements. All children younger than 11 years old gave oral consent. Written consent was given by children aged 11 or older and by a parent of all children. The study was approved by the local ethics committee of the Canton of Zurich and neighboring Cantons in Switzerland and the participants received vouchers and presents for participation.

### 2.2 Neurocognitive assessments

Extensive neurocognitive testing was performed at all time points (see Chapter 5.2 with Suppl. Tables 2 and 3). Reading fluency was assessed with word and pseudoword reading fluency scores of the Salzburger Lese- und Rechtschreibtest II (SLRT-II, (Moll and Landerl, 2010)) at T5 for the longitudinal sample and at the latest available time point for the mixed longitudinal and cross-sectional sample. Percentile scores of the SLRT-II served as standardized reading values at the chosen time point. The average percentile scores of the word and pseudoword reading fluency were log transformed with basis 2 and were entered as a covariate of interest in all our core statistical models to account for variability in reading skills. The log transformation served to approximate the covariate of average reading fluency percentile scores to a normal distribution. For a better illustration of the dimensional effect of reading skills on brain activation, children with an average percentile score of below 16 (n = 37) were considered as poor readers and children with a mean percentile score of above 25 (n = 55) as typical readers (in the respective figures the group means have been colored in red and blue). Non-verbal IQ was measured using the Reynolds Intellectual Assessment Scales (RIAS, (Reynolds and Kamphaus, 2003)) at T5 or, if not available, with the CFT1-R (Weiß and Osterland, 2013), measured between T3 and T4.

### 2.3 Experimental design

**Fig. 1:**
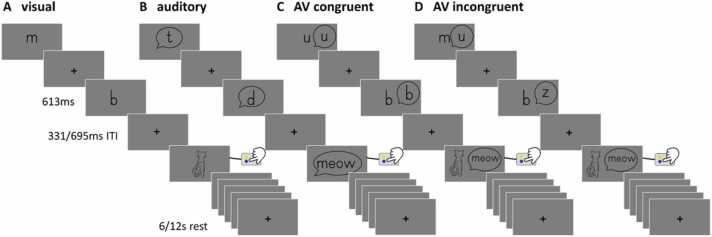
Implicit target detection task with examples of the four conditions – A) visual, B) auditory, C) audiovisual congruent and D) audiovisual incongruent. Children were instructed to press a button whenever the target (drawing or sound of a cat) appeared on the screen. In total, 216 stimuli were presented, 54 per condition. The trials were structured in blocks, with 15 trials per block and four blocks per condition, including target stimuli. The trials started with the stimulus (613 ms) followed by inter stimulus interval (331/695 ms). Resting periods of 6 s or 12 s were inserted between blocks. Abbreviations: AV, audiovisual.

During five neuroimaging sessions, we examined participants with an audiovisual target detection task as reported in Fraga-González et al. (2021), Karipidis et al. (2018), Karipidis et al. (2021), Karipidis et al. (2017), and Pleisch, Karipidis, Brauchli et al. (2019), adapted from Blau et al., 2009, Kronschnabel et al., 2014. The task included separate parts with falsefonts, letters, digits and/or short nonwords, depending on the reading level at each time point. The current study focuses on the analyses of the letter part, which comprised six letters and their corresponding speech sounds, presented in a unimodal auditory, unimodal visual, and bimodal congruent and incongruent fashion using Presentation software (www.neurobs.com). Participants were instructed to press a button, whenever the target stimulus (picture or sound of a cat, unimodal or bimodal) was presented. The analyses of only bimodal stimuli at the beginning of reading instruction, is summarized in Karipidis et al. (2021) and the corresponding EEG data of visual letter processing in Fraga-González et al. (2021). Trials were presented pseudorandomly in blocks with 15 stimuli each, including target stimuli. Blocks were separated by fixation periods of 6 or 12 s. Unimodal and bimodal blocks alternated pseudorandomly. Each condition (visual, auditory, audiovisual congruent, audiovisual incongruent) consisted of 54 stimuli and six targets, presented for 613 ms, followed by an interstimulus interval of 331 ms or 695 ms (see Fig. 1). The four conditions consisted of four blocks each. The total task duration resulted in 375 s.

The letters b, d, m, t, u, and z were used as stimuli, presented in the Swiss school font. Visual stimuli were presented via video goggles (VisuaStimDigital, Resonance Technology, Northride, CA) in black font in the middle of a grey background (RGB 128, 128, 128; mean visual angle: 2.88 horizontally; 4.88 vertically). Auditory stimuli were spoken by a female speaker, digitally recorded (sampling rate 44.1 kHz, 32 bit), and normalized in Audacity® ( ± 1 dB). They were presented over in-ear headphones (MR confon GmbH, Magdeburg). A SofTone implementation in the MRI sequence, a sound-absorbing mat in the MRI bore, and sound-absorbing over-ear headphones padded with foam material minimized acoustic noise of the MRI.

### 2.4 MRI data acquisition and preprocessing

MRI data was acquired in a simultaneous EEG-fMRI session on a Philips Achieva 3 Tesla scanner (Best, The Netherlands) using a 32-channel coil. Here, we present the fMRI data (for analyses including EEG data see Fraga-González et al., 2021, Fraga-González et al., 2022, Pleisch, Karipidis, Brauchli et al., 2019 and Karipidis et al., 2017). T2 * weighted functional images were acquired with a whole-brain gradient echoplanar image (EPI) sequence (five dummy scans followed by 189 dynamic scans, repetition time TR = 1.98 s, echo time TE = 30 ms, 31 slices, voxel size = 3.0 ×3.0 ×3.5 mm^3^, slice gap = 0.5 mm, matrix size 80 ×80 px, flip angle = 80°, sofTone factor = 3, sensitivity- encoding (SENSE) reduction factor = 2.2). For coregistration, T1 weighted images were recorded at the end of the session (TR = 6.8 ms, TE = 3.2 ms, aligned at AC-PC, flip angle = 9°, voxel size = 1.0 ×1.0 ×1.0 mm^3^, field of view = 270 ×255 mm^2^, number of slices = 176).

MATLAB (version R2017a) toolbox SPM12 (7219) was used for preprocessing and analysis, which included slice-time correction, realignment, coregistration and segmentation. Using the Template-O-Matic toolbox (Wilke et al., 2008), a pediatric template was created for normalization (average structural data with the respective mean age at each time point). Voxels were resampled to 3 × 3×3 mm^3^ isotropic voxels. An 8-mm full width at half maximum (FWHM) Gaussian kernel was applied for smoothing. After preprocessing, we repaired volumes with scan-to-scan motion above 1.5 mm with the ArtRepair toolbox (Mazaika et al., 2007), using linear interpolation between the nearest unrepaired scans. After this, we flagged volumes with and without movement surrounded by scans with motion above 1.5 mm. From the longitudinal sample of 19 children with data at each time point, three subjects with more than 20% of repaired and flagged volumes at one or more time points were not included in the final longitudinal sample of 16 children. For the mixed longitudinal and cross-sectional sample, a strict threshold of 15% repaired and flagged volumes was applied, since the analysis method of linear mixed models can handle missing values. With this threshold, data of four subjects at one time point were not included in the final mixed longitudinal and cross-sectional sample. The slightly more lenient threshold in the longitudinal design was applied to include one more subject with a percentage of repaired and flagged volumes of 17.5% at one time point. The flagged scans were scrubbed by modeling them in a binary regressor of no interest (see Supplementary Material, chapter 5.1 for details).

### 2.5 Statistical analyses

#### 2.5.1 Whole-brain analysis

For the first-level analysis, we built a general linear model using the individual onsets of six parameters of interest (auditory, visual and audiovisual congruent and incongruent trials, targets and responses) convolved with the canonical hemodynamic response function implemented in SPM12. The model also included six realignment parameters and an additional regressor of no interest with the flagged scans whenever available.

Whole-brain second-level analyses were calculated for the longitudinal sample. For the unimodal conditions, a 5 × 1 factor repeated measures analysis of variance (rmANOVA) was calculated to investigate the main effect of time point for the auditory and the visual condition separately. For the audiovisual conditions, a 5 × 2 factor rmANOVA was calculated to investigate the interaction of time point (T1, T2, T3, T4, T5) and condition (audiovisual congruent and incongruent). In a second step, we repeated the same analyses (5 ×1 rmANOVA for the visual and the auditory conditions and 5 ×2 rmANOVA for the audiovisual condition) including the log transformed covariate of reading fluency (percentile scores). We report results using a cluster-based family-wise error corrected (FWE_corr_) threshold of *p* < .05 with a cluster-defining threshold of *p* < .001.

#### 2.5.2 ROI analysis

**Fig. 2:**
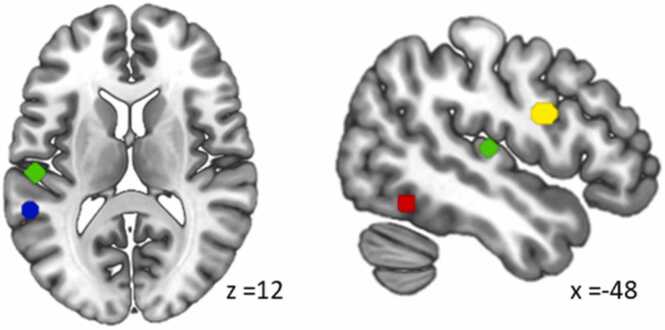
Regions of interest. Depicted are the spherical ROIs in the ventral occipitotemporal cortex (vOTC) in red, middle and posterior superior temporal gyrus (mSTG and pSTG) in green and blue and the inferior frontal gyrus (IFG) in yellow.

To investigate the developmental trajectories of letter and speech sound processing in relevant regions of the reading network, we performed region of interest (ROI) analyses using SPSS (Version 26.0, Armonk, NY: IBM Corp.). As with our previous study (Karipidis et al., 2021), ROIs were selected using the meta-analysis tool NeuroSynth (Yarkoni et al., 2011). The search term “letter” yielded two peaks, one in the left IFG (x = −47, y = 3, z = 24; upper part of pars opercularis, next to precentral gyrus) and one in the left vOTC (x = −44, y = −57, z = −15), close to the classic visual word form area (Cohen et al., 2000, Dehaene et al., 2005, Lerma-Usabiaga et al., 2018). The search term “audiovisual” yielded two peaks in the left STG, one in the middle (mSTG, x = −53, y = −21, z = 9) and one in the posterior part (pSTG, x = −56, y = −42 z = 9). Each ROI was defined as a sphere around the peak coordinates provided in MNI space with a 6 mm radius (c.f. Fig. 2). To account for recent models of vOTC function suggesting that perceptual and letter processing is located in the posterior patches of the text-selective parts in vOTC, we also defined an additional ROI based on the coordinates of the 'letter form area' (LFA) as reported by Thesen et al. (2012), and present the results of this LFA (x = −40, y = −78, z = −18) in the supplementary results (Chapter 6.3). From all these ROIs, we extracted the mean beta values using an inhouse script on the SPM plugin xjview (https://www.alivelearn.net/xjview).

After extracting the mean beta values for the ROIs, we calculated an rmANOVA for each ROI for the longitudinal sample with the factor time point (T1, T2, T3, T4, T5) to assess changes with time for i) the visual, ii) the auditory and iii) the audiovisual congruent conditions separately. In order to examine audiovisual integration processes, we calculated an rmANOVA with the factor time point for the incongruency effect (difference of mean beta values between audiovisual incongruent and congruent conditions). For significant main effects and interactions, post-hoc pairwise comparisons were computed using a Sidak-correction, if not stated otherwise. We report the within subject contrast of polynomial trend analyses to explore which polynomial function fits the data reasonably well.

To examine the effect of children’s reading skills on the developmental trajectories of letter processing, we further recomputed the same rmANOVA but added the reading fluency as a covariate of interest in the ROI analyses. The covariate reading fluency comprised the log transformed average percentiles of word and pseudoword reading measured at the last time point in fifth grade (T5).

Next, we aimed to examine the effect of reading skill on the trajectories of activation in our four ROIs in more detail using all data available (longitudinal and mixed longitudinal and cross-sectional data). We calculated linear mixed models (LMM) with the factor time point (T1, T2, T3, T4, T5) and log transformed reading fluency (percentile scores) as a covariate of interest for the four ROIs separately, and for the visual, auditory and congruent conditions, as well as for the incongruency effect. To reduce the influence of outliers and extreme values, normalized (z-score) residuals exceeding a ± 3 threshold were iteratively removed. For all conditions in all the ROIs of the LMMs, Shapiro-Wilk tests and Q-Q-plots showed normal distributions of residuals (all p > 0.05). For significant main effects and interaction effects, pairwise comparisons with Sidak-corrected p-values are reported, if not stated otherwise. For the significant main effects, we also provide effect sizes (f^2^ for the LMMs, (Selya et al., 2012); partial eta square for the rmANOVAs).

## 3 Results

### 3.1 Longitudinal sample

#### 3.1.1 Whole-brain analyses

**Fig. 3:**
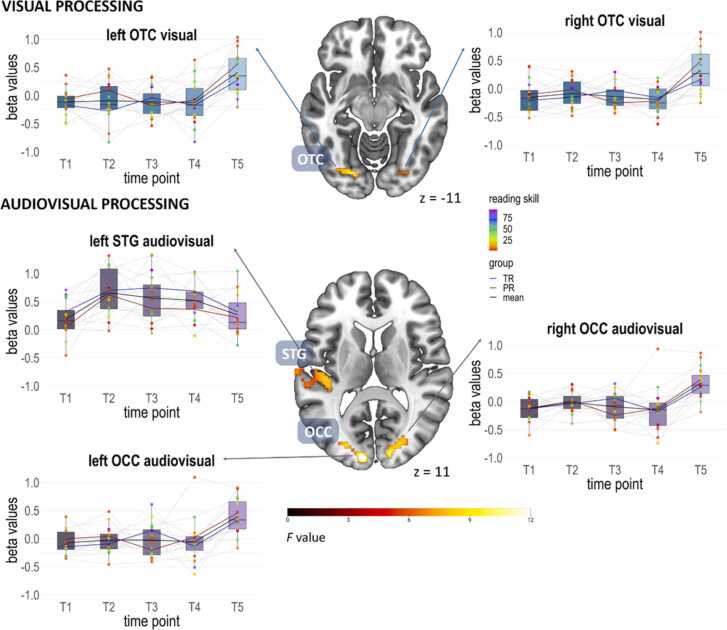
Whole brain activations (n = 16). Cluster-defining threshold p = .001. Results are cluster-level FWE corrected p < .05. The bar plots next to the slice views illustrate the individual trajectories of the beta values in the respective ROIs of the 16 children. The mean of the whole group is shown with black lines, the mean values of typical readers are shown with blue lines, mean values of poor readers are shown with red lines. Top image: Main effect of time in bilateral occipital fusiform gyri during visual processing. Bottom image: Main effect of time in bilateral occipital gyri and the superior temporal gyrus during audiovisual processing. Abbreviations: TR, typical readers; PR, poor readers; OTC, occipitotemporal cortex; OCC, occipital cortex; STG, superior temporal gyrus.

The one-way ANOVA (*n* = 16) for the unimodal visual condition with factor time point (T1, T2, T3, T4, T5) showed a significant main effect of time point in bilateral occipitotemporal regions (OT), including the fusiform gyrus, characterized by stronger blood oxygenation level-dependent (BOLD) responses in these regions at T5 compared to the other time points (c.f. Table 2 and Fig. 3). The one-way ANOVA for the unimodal auditory condition with factor time point did not yield a significant main effect of time point.

**Table 2:** Whole brain analyses (n = 16) without covariate reading fluency.

**Contrast**	**brain area**	**MNI coordinates**			**Cluster size**	**F/T-value**	**cluster-level p**_**FWEcorr**_
		**x**	**y**	**z**			
**Visual processing**							
**Main effect time point**	Fusiform gyrus L	-20	-81	-9	90	12.02	.008
	Fusiform gyrus R	19	-84	-3	106	8.75	.003
**T2 > T1**	SPL R	19	-69	42	156	3.99	.004
**T5 > T1**	OCC L	-20	-81	-6	209	5.34	.001
	OCC R	16	-93	18	164	4.29	.003
**T5 > T2**	Occipital pole L	-11	-99	3	205	5.35	.001
	OCC R	16	-87	-3	90	4.33	.042
**T5 > T3**	Fusiform gyrus L	-20	-81	-9	142	5.76	.007
	OCC R	34	-78	-6	174	4.57	.002
**T5 > T4**	Fusiform gyrus L	-20	-81	-9	172	5.76	.003
	Fusiform gyrus R	28	-78	-9	276	4.98	< .001
**Auditory processing**							
**T1 > T2**	Precuneus R	7	-66	18	218	4.41	< .001
**T2 > T5**	Heschl’s gyrus L	-38	-30	15	141	4.56	.005
**Audiovisual processing**							
**Main effect time point**	OCC L	-11	-96	9	559	15.87	< .001
	OCC R	34	-78	-9	574	14.86	< .001
	STG L	-47	-21	6	363	12.65	< .001
**T2 > T1**	PT L	-53	-24	6	548	5.50	< .001
	STG R	67	-24	0	352	5.33	< .001
**T2 > T5**	STG L	-47	-21	6	2541	5.29	< .001
**T3 > T1**	PO L	-44	-21	6	299	4.55	< .001
**T3 > T5**	Heschl’s gyrus L	-44	-21	6	138	5.62	.010
**T5 > T1**	OCC bilateral	-11	-96	9	1889	6.64	< .001
	SPL L	-26	-69	63	241	5.12	< .001
	SPL R	22	-72	54	193	4.09	.002
**T5 > T2**	OCC L	-11	-93	9	429	6.60	< .001
	OCC R	13	-93	18	330	5.39	< .001
**T5 > T3**	OCC bilateral	34	-78	-9	1075	6.18	< .001
**T5 > T4**	SOG R	31	-78	-6	877	6.43	< .001
	Occipital pole L	-8	-96	6	104	5.53	.030
	OCC L	-20	-81	-9	548	5.33	< .001

***Notes***. Cluster-defining threshold p = .001. Results are cluster-level FWE corrected p < .05. Abbreviations: OCC, occipital region; PO, parietal operculum; PT, planum temporale; SPL, superior parietal lobule; SOG, superior occipital gyrus; STG, superior temporal gyrus; L, left; R, right.

The one-way ANOVA with factors time point (T1, T2, T3, T4, T5) and congruency (congruent, incongruent) for the audiovisual processing conditions showed a significant main effect of time point in bilateral occipital regions (OCC) and the left superior temporal gyrus (STG), characterized by stronger BOLD responses at T5 than at the other time points in the OCC and stronger BOLD responses at T2 than T1 and T5 in the STG (c.f. Table 2 and Fig. 3). The one-way ANOVAs for visual, auditory, and audiovisual processing with the covariate reading fluency (log transformed percentile scores of reading fluency) produced similar results and can be found in Supplementary Table 4.

#### 3.1.2 ROI analyses

Next, we performed ROI analysis in several regions of the reading network: The vOTC, mSTG, pSTG and IFG. The statistics of all models for the different samples and ROIs are reported in the next sections and are also summarized in Table 3 for a better overview.

**Table 3:** Summary of main effects and interactions of the longitudinal and cross-sectional ROI analyses.

**ROI**	**Condition**	**Effect**	**longitudinal (no cov.);** **rmANOVA**	**longitudinal (cov. RF);** **rmANOVA**	**mixed longitudinal and cross-sectional (cov. RF); LMM**
vOTC	V	TP	**F(4,60)= 3.15, p = .020**	n.s. (p = .350)	**F(4154.96)= 4.89, p = .001**
		TP*RF	-	n.s. (p = .267)	F(4155.77)= 2.41, p = .052
		RF	-	n.s. (p = .621)	n.s. (p = .971)
	A	TP	n.s. (p = .320)	n.s. (p = .164)	F(4161.74)= 2.18, p = .073
		TP*RF	-	n.s. (p = .288)	F(4161.14)= 2.01, p = .095
		RF	-	n.s. (p = .959)	n.s. (p = .856)
	AV cong	TP	**F(4,60)= 6.09, p < .001**	n.s. (p = .286)	**F(4135.78)= 4.66, p = .001**
		TP*RF	-	n.s. (p = .232)	n.s. (p = .259)
		RF	-	n.s. (p = .907)	n.s. (p = .153)
	Inco effect	TP	n.s. (p = .627)	n.s. (p = .200)	n.s. (p = .112)
		TP*RF	-	n.s. (p = .233)	**F(4183.22)= 2.76, p = .029**
		RF	-	n.s. (p = .162)	n.s. (p = .451)
mSTG	V	TP	n.s. (p = .413)	n.s. (p = .838)	n.s. (p = .951)
		TP*RF	-	n.s. (p = .984)	n.s. (p = .316)
		RF	-	n.s. (p = .346)	n.s. (p = .816)
	A	TP	**F(4,60)= 3.83, p = .008**	n.s. (p = .387)	n.s. (p = .490)
		TP*RF	-	n.s. (p = .851)	n.s. (p = .135)
		RF	-	n.s. (p = .187)	F(1,82.69)= 3.90, p = .052
	AV cong	TP	**F(4,60)= 5.18, p = .001**	n.s. (p = .281)	n.s. (p = .680)
		TP*RF	-	n.s. (p = .826)	n.s. (p = .544)
		RF	-	F(1,14)= 4.18, p = .060	**F(1,81.86)= 4.23, p = .043**
	Inco effect	TP	n.s. (p = .744)	n.s. (p = .225)	n.s. (p = .265)
		TP*RF	-	n.s. (p = .275)	F(4193.32)= 2.03, p = .092
		RF	-	n.s. (p = .584)	n.s. (p = .348)
pSTG	V	TP	n.s. (p = .192)	n.s. (p = .712)	F(4147.07)= 2.01, p = .097
		TP*RF	-	n.s. (p = .888)	n.s. (p = .115)
		RF	-	n.s. (p = .489)	n.s. (p = .990)
	A	TP	n.s. (p = .522)	n.s. (p = .540)	**F(4145.19)= 3.08, p = .018**
		TP*RF	-	n.s. (p = .514)	**F(4146.72)= 3.15, p = .016**
		RF	-	**F(1,14)= 6.02, p = .028**	n.s. (p = .177)
	AV cong	TP	n.s. (p = .196)	n.s. (p = .685)	n.s. (p = .102)
		TP*RF	-	n.s. (p = .485)	**F(4155.42)= 3.23, p = .014**
		RF	-	n.s. (p = .182)	**F(1101.82)= 4.35, p = .039**
	Inco effect	TP	n.s. (p = .334)	n.s. (p = .800)	F(4191.41)= 2.37, p = .054
		TP*RF	-	n.s. (p = .707)	**F(4190.67)= 2.54, p = .042**
		RF	-	n.s. (p = .410)	n.s. (p = .951)
IFG	V	TP	n.s. (p = .553)	n.s. (p = .712)	n.s. (p = .393)
		TP*RF	-	n.s. (p = .814)	n.s. (p = .265)
		RF	-	n.s. (p = .546)	n.s. (p = .372)
	A	TP	n.s. (p = .681)	n.s. (p = .176)	**F(4168.74)= 3.30, p = .012**
		TP*RF	-	n.s. (p = .223)	F(4168.31)= 2.35, p = .056
		RF	-	n.s. (p = .294)	n.s. (p = .335)
	AV cong	TP	**F(4,60)= 2.58, p = .047**	n.s. (p = .289)	n.s. (p = .115)
		TP*RF	-	n.s. (p = .661)	n.s. (p = .512)
		RF	-	F(1,14)= 3.13, p = .098	**F(1,61.79)= 9.45**, **p = .003**
	Inco effect	TP	F(4,60)= 2.11, p = .091	**F(4,56)= 3.57, p = .012**	**F(4162.48)= 2.84, p = .026**
		TP*RF	-	**F(4,56)= 3.10, p = .022**	n.s. (p = .103)
		RF	-	n.s. (p = .776)	n.s. (p = .969)

*Notes*. List of main effects and interactions for the different models: Longitudinal analyses without covariate (analyzed with an rmANOVA), longitudinal analyses with covariate reading fluency (analyzed with an rmANOVA) and mixed longitudinal/cross-sectional (analyzed with an LMM). Significant effects and interactions of p < .05 are printed in bold, trends are printed in regular font. Abbreviations: rmANOVA, repeated measures analysis of variance; LMM, linear mixed model; ROI, region of interest; cov, covariate; TP, time point; RF, reading fluency (log transformed percentile scores); vOTC, visual occipitotemporal cortex; mSTG, middle superior temporal gyrus; pSTG, posterior superior temporal gyrus; IFG, inferior frontal gyrus; V, visual condition; A, auditory condition; AV cong, audiovisual congruent condition; inco effect, incongruency effect.

First, we were interested in overall developmental patterns of activation in the four ROIs of our longitudinal sample (n = 16). In the left vOTC ROI, there were significant main effects of time point for the visual (*F*(4,60)= 3.15, *p* = .020, η_p_^2^ = 0.174) and the congruent conditions (*F*(4,60)= 6.09, *p* < .001, η_p_^2^ = 0.289), but not for the auditory condition (*p* = .320). Both main effects of time point showed a cubic trend (*F*(1,15)= 6.37, *p* = .023 for the visual condition; *F*(1,15)= 8.56, *p* = .010 for the congruent condition), the congruent condition additionally showed a linear trend (*F*(1,15)= 13.21, *p* = .002). Sidak-corrected pairwise comparisons showed a trend for a stronger activation at T5 than T4 (*p*_*corr*_=.083), but no other significant differences between time points for the visual condition (*p*s ≥ .120); uncorrected pairwise comparisons showed significantly stronger activations at T5 than T1 (*p*_*unc*_=.013) and than T4 (*p*_*unc*_=.009) and trends for stronger activations at T3 than T1 (*p*_*unc*_=.066) and T4 (*p*_*unc*_=.065), which possibly explain the cubic trend in the time effect. Sidak-corrected pairwise comparisons for the congruent condition showed stronger activations at T5 than T1 (*p*_*corr*_=.002), than T2 (*p*_*corr*_=.044), and than T4 (*p*_*corr*_=.030).

In the left mSTG ROI, there were significant main effects of time point for the auditory (*F*(4,60)= 3.83, *p* = .008, η_p_^2^ = 0.203) and the congruent conditions (*F*(4,60)= 5.18, *p* = .001, η_p_^2^ = 0.257), but not the visual condition (*p* = .413). The main effects showed a quadratic trend (*F*(1,15)= 16.09, *p* = .001 for the auditory condition; *F*(1,15)= 22.21) *p* < .001 for the congruent condition). Sidak-corrected pairwise comparisons for the auditory condition showed increasing activations from T1 to T2 (*p*_*corr*_=.034) and a trend for decreasing activations from T2 to T5 (*p*_*corr*_=.063). Sidak-corrected pairwise comparisons for the congruent condition showed increasing activations from T1 to T2 (*p*_*corr*_=.020), and from T1 to T3 (*p*_*corr*_=.016). In the left pSTG ROI, we found no significant effects of time point in any of the conditions (*p*s > .192).

**Fig. 4:**
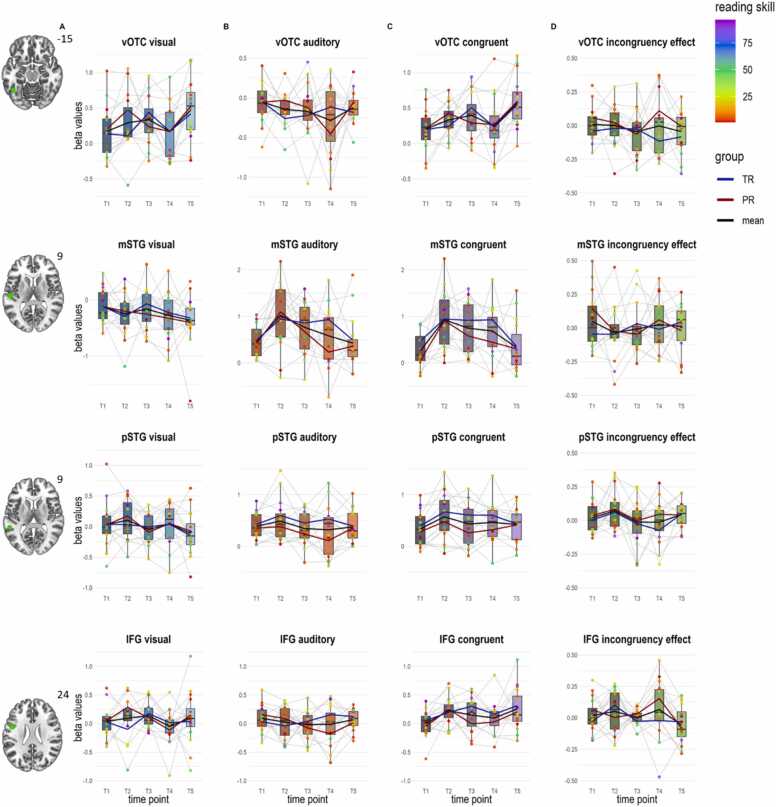
Results of the ROI analyses for the longitudinal sample within the vOTC, mSTG, pSTG and IFG ROI (top to bottom, numbers next to slice views refer to the MNI z coordinates). Depicted are the beta values extracted A) during visual letter processing B) during auditory speech sound processing, C) during congruent audiovisual processing, and D) for the audiovisual incongruency effect per time point. T1: kindergarten; T2: Middle of 1st grade; T3: End of 1st grade; T4: Middle of 2nd grade; T5: Middle of 5th grade. Developmental trajectories of activation are shown in black for the whole sample and separately for children considered as typical readers (blue line) or poor readers (red line), colored dots represent single subjects, and the colors reflect individual reading skill percentiles as indicated by the color bar. Abbreviations: ROI, region of interest; vOTC, ventral occipitotemporal cortex; mSTG, middle superior temporal gyrus; pSTG, posterior superior temporal gyrus; IFG, inferior frontal gyrus; TR, typical readers; PR, poor readers.

In the left IFG, there was a significant main effect of time point for the congruent condition only (*F*(4,60)= 2.58, *p* = .047, η_p_^2^ = 0.146). This effect showed a cubic trend (*F*(1,15)= 4.71, *p* = .046) and Sidak-corrected pairwise comparisons showed a trend for stronger activations at T2 than T1 (*p*_*corr*_=.082). The main effect of time point in the IFG was at a trend level when modeling the incongruency effect (inco-cong, *F*(4,60)= 2.11, *p* = .091, η_p_^2^ = 0.123), and no significant main effect of time point was found in any of the other ROIs for the incongruency effect (*p*s > .334). We present the trajectories of the longitudinal sample in Fig. 4 for the results in the vOTC, mSTG, pSTG and IFG.

Due to the high variability and broad range of children’s reading scores, next, we added log transformed T5 reading fluency percentile scores as a covariate of interest to the longitudinal analysis using rmANOVAs. The addition of this covariate changed the results. First, there were no significant main effects or interactions in the vOTC (*p*s > .164). In the two STG ROIs, there was a significant (pSTG: *F*(1,14)= 6.02, *p* = .028, η_p_^2^ = 0.301) and trend level (mSTG: *F*(1,14)= 4.18, *p* = .060, η_p_^2^ = 0.230) effect of reading fluency on the activation of the auditory (pSTG) and the audiovisual (mSTG) condition, with better readers showing stronger beta values in the ROI than poorer readers. The main effects of time point did not exceed significance levels in any of the ROIs and conditions (visual, auditory or congruent audiovisual) (*p*s > .164), except for the incongruency effect in the IFG (*F*(4,56)= 3.57, *p* = .012, η_p_^2^ = 0.203). This effect showed a cubic trend (*F*(1,14)= 12.38, *p* = .003), indicating a cubic trajectory in activation strength of the IFG over time when controlling for reading fluency. Sidak-corrected pairwise comparisons for the IFG showed no significant differences between single time points (*p*s > .154), uncorrected comparisons showed a significantly stronger incongruency effect at T1 than T5 (*p*_*unc*_=.030) and at T4 than T5 (*p*_*unc.*_=.017). Accounting for the covariate reading fluency provided some interesting insights into potential modulations of activation trajectories through reading skills. There was a significant interaction of time point and reading fluency in the IFG for the incongruency effect (*F*(4,56)= 3.10, *p* = .022) with a cubic trend (*F*(1,14)= 11.48, *p* = .004), which may indicate a tendency towards an earlier peak of the incongruency effect in better readers (T2) as compared with poor readers (T4). There were no other significant effects of the covariate reading fluency or interactions between reading fluency and time point (*p*s > .162).

The pronounced difference in the above reported models when including reading fluency as a covariate, highlighted the need for a better understanding of the effect of reading skills on the activation trajectories in our ROIs. Because the fully longitudinal sample was too small to further explore such potential differences, we continued with examining the pooled longitudinal and cross-sectional data of our study as detailed in the next section.

### 3.2 Mixed longitudinal and cross-sectional sample (n = 102)

**Fig. 5:**
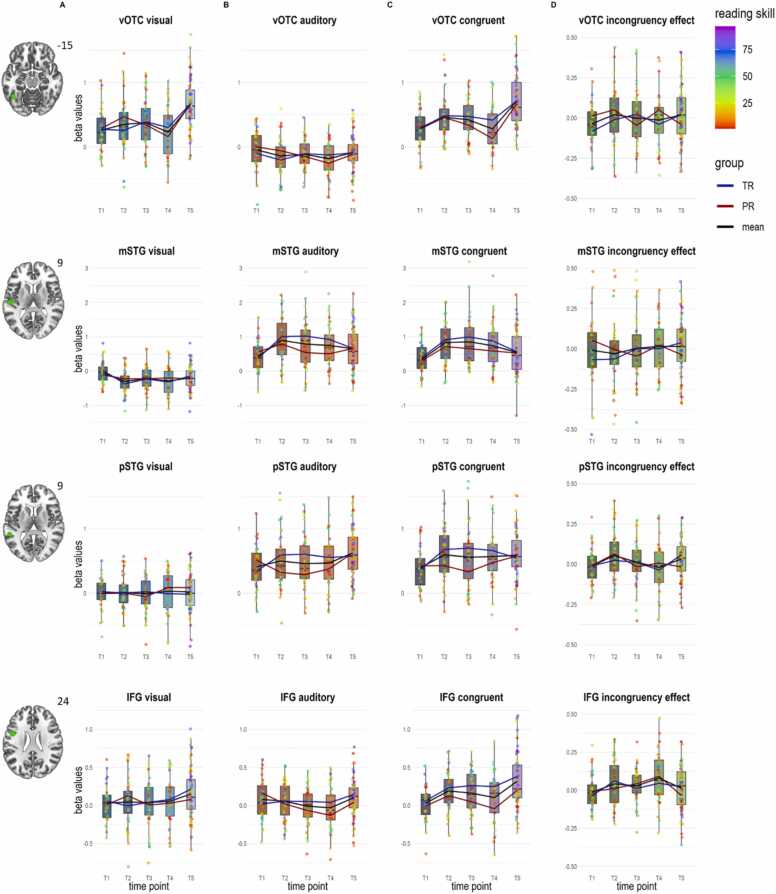
Results of the ROI analyses for the pooled longitudinal and cross-sectional sample within the vOTC, mSTG, pSTG and IFG ROI (top to bottom, numbers next to slice views refer to the MNI z coordinates). Depicted are the beta values extracted A) during visual letter processing B) during auditory speech sound processing, C) during congruent audiovisual processing, and D) for the audiovisual incongruency effect per time point. T1: kindergarten; T2: Middle of 1st grade; T3: End of 1st grade; T4: Middle of 2nd grade; T5: Middle of 5th grade. Developmental trajectories of activation are shown in black for the whole sample and separately for children considered as typical readers (blue line) or poor readers (red line), colored dots represent single subjects, and the colors reflect individual reading skill percentiles as indicated by the color bar. Abbreviations: ROI, region of interest; vOTC, ventral occipitotemporal cortex; mSTG, middle superior temporal gyrus; pSTG, posterior superior temporal gyrus; IFG, inferior frontal gyrus; TR, typical readers; PR, poor readers.

In the *left vOTC ROI* (Fig. 5, 1st row), our LMMs with factor time point and the covariate reading fluency revealed a significant main effect of time point for the visual condition (*F*(4154.96)= 4.89, *p* = .001, f^2^ = 0.211, Fig. 5a), with stronger activation at T5 than T1, T2, T3 and T4 (*p*s_*corr*_≤.001, uncorrected comparisons showed an additional trend for a stronger activation at T3 than T4 (*p*_*unc*_=.060)), and a trend for the interaction of time point and reading fluency (*F*(4155.77)= 2.41, *p* = .052), but no significant effect of reading fluency (*p* = .971). There was a significant main effect of time point for the congruent condition (*F*(4135.78)= 4.66, *p* = .001, f^2^ = 0.241, Fig. 5c), with a stronger activation at T5 than T1, T2, T3 and T4 (*p*s_*corr*_≤.002), but no significant effect of reading fluency or interaction of time point and the covariate (*p*s > .153). Finally, we found a significant interaction of reading fluency and time point for the incongruency effect (*F*(4183.22)= 2.76, *p* = .029, Fig. 5d), but no significant effect of reading fluency or time point (*p*s > .112). For the auditory condition, main effects and interaction effect between time point and reading fluency did not reach significance (*p*s > .073, Table 3).

In the *left mSTG ROI* (Fig. 5, 2nd row), we found a significant effect of reading fluency for the model of the congruent condition (*F*(1,81.86)= 4.23, *p* = .043, f^2^ < −0.001, Fig. 5c), supporting the previously reported trend seen in the longitudinal sample and characterized by stronger beta values with better reading skills for audiovisual processing. There were no significant main effects of time point or interaction between time point and reading fluency for the congruent condition (*p*s > .544). For all the other conditions, we found no significant main effects or interactions (*p*s > .135). However, there was a trend for the effect of reading fluency in the auditory condition (*F*(1,82.69)= 3.90, *p* = .052, f^2^ = 0.020, Fig. 5b), indicating a similar effect of reading fluency on unimodal auditory processing as seen for audiovisual processing, and for the interaction of time point and reading fluency for the incongruency effect (*F*(4193.32)= 2.03, *p* = .092, Fig. 5d).

In the *left pSTG ROI* (Fig. 5, 3rd row), there was a trend for the main effect of time point for the visual condition (*F*(4147.07)= 2.01, *p* = .092, f^2^ = 0.014, Fig. 5a), but no significant effect of reading fluency or interaction for the visual condition (*p*s > .115). Furthermore, there was a significant main effect of time point for the auditory condition (*F*(4145.19)= 3.08, *p* = .018, f^2^ = 0.035, Fig. 5b). Sidak-corrected pairwise comparisons showed no significant differences between single time points (*p*s > .455), uncorrected comparisons showed a trend for a stronger activation at T5 than T1 (*p*_*unc*_=.059). For the auditory condition, we additionally found a significant interaction of time point and reading fluency (*F*(4146.72)= 3.15, *p* = .016). Further, we found a significant effect of reading fluency (*F*(1101.82)= 4.35, *p* = .039, f^2^ = 0.066), indicating higher beta values with better reading skills and a significant interaction of reading fluency and time point for the congruent condition (*F*(4155.42)= 3.23, *p* = .014, Fig. 5c), but no significant main effect of time point (*p* = .102). The interactions of reading fluency with time point in the left pSTG for both the auditory and congruent audiovisual conditions can best be described as revealing different, nonlinear activation curves in better (inverse U-shape) versus poorer (U-shape) readers. Lastly, there was a trend for an effect of time point (*p* = .054, f^2^ =0.051), and significant interaction of time point and reading fluency for the incongruency effect (*F*(4190.67)= 2.54, *p* = .042, Fig. 5d).

In the *left IFG ROI* (Fig. 5, 4th row), there were no significant main effects or interaction for the visual condition (*p*s > .265). There was a significant main effect of time point for the auditory condition (*F*(4168.74)= 3.30, *p* = .012, f^2^ = 0.059, Fig. 5b). Sidak-corrected pairwise comparisons showed no significant differences between time points (*p*s ≥ .122), uncorrected comparisons showed stronger activation at T5 compared to T3 (*p*_*unc*_=.018) and T4 (*p*_*unc*_=.013). Furthermore, for the auditory condition, there was a trend for the interaction of time point and reading fluency (*F*(4168.31)= 2.35, *p* = .056). We found a significant effect of reading fluency for processing the congruent condition (*F*(1,61.79)= 9.45, *p* = .003, f^2^ = 0.038, Fig. 5c), indicating higher beta values in better readers, but no significant main effect of time point or interaction effect (*p*s > .115). Finally, we found a significant main effect of time point for the incongruency effect (*F*(4162.48)= 2.84, *p* = .026, f^2^ = 0.082, Fig. 5d), with a stronger incongruency effect at T4 than T1 (*p*_*corr*_=.017).

In summary, we found an inverted U-shaped development of auditory and audiovisual processing of letters and speech sounds in the superior temporal cortex, as seen in the mSTG ROI and the whole-brain analyses. Visual and audiovisual processing of letters and speech sounds in the occipitotemporal cortex was characterized by a cubic developmental trajectory that did not significantly depend on reading fluency. Furthermore, there was a significant effect of reading skills on activation in the IFG and both STG ROIs during audiovisual processing, characterized by an increased activation for children with higher reading skills during the period of reading acquisition (T2-T5). Lastly, there was a significant interaction of reading skills and time point for the incongruency effect in the STG and vOTC.

## 4 Discussion

In this fMRI study, we investigated the developmental trajectories of visual, auditory, and audiovisual letter–speech-sound processing and integration from a prereading stage through an early reading stage to advanced reading. We analyzed data from a small but fully longitudinal sample and data from a large, mixed longitudinal and cross-sectional sample of children in kindergarten, before reading acquisition (T1), in the middle of first grade (T2), at the end of first grade (T3), in the middle of second grade (T4) and at the end of formal reading instruction in fifth grade (T5). Our analyses yielded three main findings. First, we found significant developmental changes in cortical activation to print and speech sound processing in left-hemispheric regions of the reading network involved in processing speech, written text, and audiovisual integration such as the superior temporal gyrus (STG), the ventral occipitotemporal cortex (vOTC) and the inferior frontal gyrus (IFG). Second, reading skills modulate the level of activation mainly in left temporal and frontal areas when processing congruent audiovisual letter–speech sound information. Reading skills also had an effect on functional activation development, characterized by attenuated (middle STG, mSTG) or even inverse (posterior STG, pSTG) trajectories in children with poor reading skills. Third, the effect of reading fluency on audiovisual integration in the posterior STG and vOTC, measured by the incongruency effect, depended on the time point.

### 4.1 Developmental trajectories of letter and speech sound processing during reading acquisition

The whole-brain analyses of our longitudinal sample suggested that brain activation to unimodal visual and bimodal audiovisual processing of letters changes in different regions of the reading network over the course of reading acquisition (Richlan, 2019). Our results showed a main effect of time point in the occipitotemporal cortex during visual processing and in the superior temporal gyrus and occipital cortex during audiovisual processing. Further analyses indicated that this developmental effect was mainly driven by an increase in activation in the occipital and occipitotemporal regions towards the end of elementary school in fifth grade, thus at an advanced stage of reading instruction, for both visual and audiovisual letter processing. In contrast, the main effect of time point in the superior temporal gyrus was an increase in activation within auditory regions during intense reading instruction in second grade and a subsequent decrease in activation at the end of formal reading instruction in fifth grade, thus following an inverted U-shaped developmental trajectory. Subsequent region of interest (ROI) analyses in the longitudinal and the large mixed longitudinal and cross-sectional samples clarified the trajectories detected in our whole-brain results.

The vOTC activation showed a complex trajectory for both the unimodal visual and the audiovisual congruent condition. This trajectory of the activation in the left vOTC ROI, located around the VWFA, indicates an increase in response to print at the beginning of formal reading instruction as has been described in other studies (Ben-Shachar et al., 2011, Brem et al., 2010, Centanni et al., 2017, Dehaene-Lambertz et al., 2018, Fraga-González et al., 2021, Maurer et al., 2006). This is also in line with studies that have shown the VWFA to respond increasingly to letter-like characters after brief artificial letter training, confirming that audiovisual letter–speech-sound learning drives visual specialization in the vOTC (Pleisch, Karipidis, Brauchli et al., 2019).

After the initial increase in activation at the beginning of primary school, a second, much more pronounced activation peak was found at the end of primary school. The increase in visual vOTC response to letters in fifth grade was unexpected for two reasons. Firstly, our previous analysis of EEG data from a largely overlapping sample in the same study showed that the inverted U-shaped development of visual sensitivity to letters extended to fifth grade (Fraga-González et al., 2021), as measured with in the electrophysiological counterpart of vOTC activation, the visual N1 event-related potential (ERP) activity (Hirshorn et al., 2016). This trajectory of visual N1 activation to letters corresponded to ERP findings of previous studies, such as the inverted U-shaped developmental trajectories reported for visual N1 word and letter-string processing (Maurer et al., 2006, Maurer et al., 2011, Park et al., 2018, Zhao et al., 2019). Although these ERP studies focused on sensitivity to print by comparing letters or words with falsefonts and falsefont strings, amplitude changes to letters and words also showed a similar inverted U shape development. Secondly, fMRI findings also suggested an inverted U-shaped development in several regions within the reading network, such as the VWFA, the posterior parietal cortex, and the inferior frontal cortex, with a peak of activation at the onset of schooling followed by a slight decline (Dehaene-Lambertz et al., 2018). Despite the methodological challenge of directly comparing more rapid and transient EEG data with prolonged, re-entrant, and sustained fMRI data, the literature so far was rather consistent in characterizing developmental trajectories for visual letter and letter string processing in vOTC and its electrophysiological correlate the visual N1 ERP. It is therefore possible that the BOLD response measured in the vOTC ROI reflects not only early text recognition processes (150–250 ms) but also later processes (300–500 ms) that are assumed to develop with increasing reading expertise (Caffarra, Karipidis et al., 2021). However, large and fully longitudinal assessments of the vOTC BOLD response to processing letters or words over the period of elementary school are still lacking.

Our finding of a pronounced increase in activation between second grade and the more advanced reading stage in fifth grade might be explained in several ways. Firstly, we presented letters in isolation and not within strings or words, as was the case in most previous studies (Brem et al., 2010, Dehaene-Lambertz et al., 2018, Maurer et al., 2006, Maurer et al., 2011, Pleisch et al., 2019, Saygin et al., 2016). Future studies are needed to compare the developmental changes in the audiovisual activation of single letter and word or letter-string processing directly to better understand how reading expertise and age modulate their distinct trajectories. The activation to single letters may vary with the varying contexts of processing at distinct time points during the learning procedure and may also depend on reading expertise. When children learn to read in transparent alphabetic languages, they first start processing letters as a separate entity by linking letters to speech sounds. After familiarization and with growing automatization of letter–speech-sound associations, letters are mostly processed in larger units such as syllables, morphemes, words, and sentences. Later on, single letters are also used in abbreviations or as variables in algebra. A change in a child’s learning context might thus induce a shift in the processing of letters. This may include differences in attention to single letters in different phases of learning. As previous evidence also points to the VWFA as a gateway linking language and attention systems (Chen et al., 2019, Yeatman et al., 2014), shifts in strategy and attention when processing single letters could help to explain the cubic developmental trajectory. Secondly, considering the rather long interval of three years between measurements taken in second grade at mean age 8.4 and fifth grade at mean age 11.5, we expect pronounced maturation processes shaping brain structure and connectivity in the young brain. In particular, the word-sensitive regions in the left vOTC show increases in functional activation volume and categorical selectivity to words from young childhood to teenage years (Ben-Shachar et al., 2011, Nordt et al., 2021). Consequently, the rapid increase in visual activation in the vOTC could also be explained by such an expansion of the activation volume to letters as categorical specialization proceeds. Another effect of maturation also involves the changes in connectivity among relevant brain regions (Cao et al., 2018, Horwitz et al., 1998). This may include alterations in structural and functional connections to other regions of both the language network and the attentional system (Bitan et al., 2009, Chen et al., 2019, Di Pietro et al., 2023, Morken et al., 2017, Takemura et al., 2016, Weiner et al., 2017, Yeatman et al., 2014). Such changes in connectivity and the associated changes in feedback processing in vOTC could modulate the activation measured.

Next, the vOTC ROI definition, as determined by Neurosynth, was found to be close to the classic VWFA. Anterior vOTC regions have been associated with linguistic and lexical aspects of word processing, and more posterior regions have been implicated in perceptual processes and letter-form processing (Lerma-Usabiaga et al., 2018, Thesen et al., 2012). To address a possible difference in the functional development of anterior and posterior vOTC regions, we also report and visualize the trajectory of the left posterior vOTC patch, which covers the previously identified "letter form area" (Thesen et al., 2012). The results presented in the supplementary material suggest similar developmental trajectories of the posterior and anterior vOTC activations. Posterior vOTC activation showed a steep increase by fifth grade during visual and audiovisual processing, similarly to the anterior vOTC patch. Finally, despite the stringent motion correction criteria applied in our study, the higher motion-induced artefacts in younger children than older ones, which we expected, may also contribute to an increase in the signal-to-noise ratio with age. However, no similar steep signal increases from T4 to T5 were found in other ROIs, which indicates that the activation changes cannot solely be explained by differences in motion. In sum, a better understanding of the rather unexpected steep increase in visual activation within the vOTC ROI needs further investigation, such as additional follow-ups at later ages and the examination of alterations in functional connectivity and/or neuroanatomical measures, including grey and white matter volumes within these circuits. Whether a further phase of activation decrease due to expertise and refinement in the underlying neural network may follow after fifth grade cannot be answered with the current data but needs additional longitudinal assessments through adolescence to adulthood.

Reading instruction increases activation within phonological processing areas, such as the superior temporal cortex (Monzalvo and Dehaene-Lambertz, 2013), and its activation scales with individual differences in phonological (Bonte et al., 2016) and reading skills (Brennan et al., 2013). Similarly to our previous study with a partially overlapping sample (Karipidis et al., 2021), we found an increase in left STG activation to audiovisual congruent letter–speech-sound pairs in the whole-brain analysis after just a few months of reading instruction, followed by a decrease towards the end of primary school. In our subsequent ROI analyses, similar trajectories were found in the mSTG for the auditory and audiovisual conditions in the longitudinal sample and the pSTG for the auditory condition in the mixed longitudinal and cross-sectional sample. The inverse U-shaped trajectories suggest a reduced involvement of auditory processing areas with more automatized reading and refinements in speech processing. In Romanovska et al.'s (2022) study, children aged 8–11 years were repeatedly measured with a text-based recalibration paradigm in an fMRI study over three time points. Activation during audiovisual processing showed an inverted U-shaped development within the STG and the lateral precentral gyri for children aged 8 years at the first measurement and a linear development for children aged 9 years at the first measurement. Whereas we observed the peak of activation in the STG at around 7.5 years, the peak in activation reported by Romanovska et al. (2022) was at a later age, 9.8 years. It is likely that this discrepancy can be explained by the methodological differences between the two studies. For example, the stimulus type was more complex in Romanovska et al.'s (2022) study, because participants were presented with two trigrams, “aba” or “ada”, whereas our study presented single letters and speech sounds. Our ERP data suggest that implicit letter processing might show an earlier activation peak than word processing due to its faster automatization process in the visual domain (Fraga-González et al., 2021); whether this also applies to the auditory domain needs further research.

### 4.2 Influence of reading skills on brain activation during letter and speech-sound processing

In addition to the occipitotemporal and the superior temporal regions, frontal and parietal regions have been shown to contribute to the reading process (Pugh et al., 2000, Sandak et al., 2004, Zhou et al., 2016). The convergent activation of speech and print onto a common fronto-temporo-parietal network across different languages and writing systems has suggested that it is a universal signature of proficient reading (Rueckl et al., 2015) that starts to develop with reading acquisition (Chyl et al., 2018), is associated with the level of phonological awareness (Frost et al., 2009), and is predictive of future reading achievements (Preston et al., 2016). Besides its relevance to phonological and articulatory coding for speech and print (Bach et al., 2010, Preston et al., 2016, Price, 2012), the IFG is also considered an important region for multisensory integration, especially in speech perception (McCormick et al., 2018, Moris Fernandez et al., 2017, Tse et al., 2015) and multimodal information categorization (Li et al., 2020). We report a significant effect of reading skill on activation during congruent audiovisual processing in the IFG and the middle and posterior STG ROIs; this result shows that, on average, typical readers activated these regions more than poor readers independent of their age. This in turn indicates deficient audiovisual processing of letters and speech sounds in poor readers in multisensory integration areas such as the IFG and the STG. This is in accordance with previous studies that show altered activation to print in children with dyslexia than typically developing children in the inferior frontal region (Brem et al., 2010, Chyl et al., 2019, Richlan, 2019, Richlan et al., 2009) and a positive correlation between word reading skills and STS activation during print and speech processing (Chyl et al., 2018). Further evidence comes from a study showing that lesions in the STS hinder audiovisual integration in adults (Hickok et al., 2018) and from studies reporting diminished letter–speech-sound integration in the superior temporal region in adults (Blau et al., 2009, Ye et al., 2017) and children (Blau et al., 2010) with dyslexia.

### 4.3 Developmental trajectories depend on reading outcome

Children’s reading skills modulated activation levels differently in several brain regions across time points. For vOTC and IFG activation, only trends were detected in response to unimodal conditions, but the pSTG activation trajectory was modulated by the reading skills of children for both the unimodal auditory condition and for the audiovisual conditions. Results from a previous study of ours showed that STG activation increases at the beginning of reading acquisition in typically reading children, whereas children who later develop poor reading skills do not show such an increase (Karipidis et al., 2021). Similarly, we found here that the developmental trajectory for children with typical reading skills resembles an inverted U shape, whereas that for poorly reading children showed an opposite trajectory with a U-shaped course, suggesting clearly different trajectories especially during the intense learning phase from kindergarten to fifth grade. The posterior STG is especially relevant to audiovisual integration (Ye et al., 2017). It is therefore possible that deficient audiovisual integration processes in poor readers at the beginning of primary school diminish activation in this region during the intense phase of letter-to-speech sound-mapping.

Previous literature showed (in)congruency effects in superior temporal regions (Blau et al., 2010, Blau et al., 2009, Caffarra et al., 2021, Kronschnabel et al., 2014, Plewko et al., 2018, van Atteveldt et al., 2004). Here, we report an interaction of the incongruency effect with reading skills and time point in the superior temporal regions and the vOTC. This finding suggests that an incongruency effect develops at different rates depending on reading skills, a suggestion also supported by our previous work showing a relationship between the incongruency effect and reading outcome (Karipidis et al., 2021). Interestingly, Plewko et al. (2018) showed that preschool children who later developed reading difficulties presented higher activations in the superior temporal cortex for audiovisually congruent stimuli than children who later developed typical reading skills. Here, we do not see clear patterns of differentiation between children with typical reading skills and those with poor reading skills. However, it seems that for the vOTC and the mSTG at kindergarten age, children with better reading outcomes showed a congruency effect, which diverges from Plewko et al.'s (2018) findings. The inverted U-shaped development of audiovisual processing in the vOTC and the cube-shaped development in STG activation were not reflected in the incongruency effect. The developmental trajectories for audiovisual processing are likely driven by the underlying unimodal processing, because the trajectory of audiovisual processing of the vOTC is similar to that of visual processing of the vOTC, but the trajectory of audiovisual processing in the STG is similar to that of auditory processing in the STG. However, we did not find developmental effects of audiovisual integration, measured with the incongruency effect, suggesting that audiovisual integration of letters and speech sounds in these regions does not change significantly with increasing reading experience. Conversely, we do report an effect of time point on the incongruency effect for the IFG, suggesting a reading-independent audiovisual integration change in this region. Future studies should confirm the aberrant developmental trajectories of STG and vOTC activation to audiovisual information and of audiovisual integration in children with dyslexia.

### 4.4 Limitations and outlook

A limitation of the interpretability and generalizability of our findings is that our longitudinal sample consisted of children with a wide range of reading skills with a non-normal distribution and an over-representation of children with poor reading skills. Nevertheless, it is important to investigate a wide variety of reading skills, because a better understanding of reading development may help to individualize interventions. To explore the interplay between reading development and letter–speech-sound processing trajectories, we included the reading skills as a covariate in our analyses. The results obtained from the longitudinal sample and the figures of subgroups of children with typical and poor reading skills require careful interpretation, especially due to the small sample size for the longitudinal analyses and the significant impact of the reading fluency covariate on the results. To examine the effect of reading fluency on the developmental trajectories in the core regions of interest in more detail, we also studied the large, mixed longitudinal and cross-sectional sample.

### 4.5 Conclusion

The longitudinal trajectories observed in children’s STG responses to auditory and audiovisual stimuli indicate a fine tuning of speech perception areas during development and reading acquisition. This inverted U-shaped development might represent a shift from effortful to automatized and refined processing of speech sounds. Furthermore, the more complex developmental trajectory of the vOTC activation to visual and audiovisual stimuli was characterized by a first peak in activation at the beginning of formal reading instruction followed by a sharp increase in activation in a more advanced reading stage. This second peak at the end of primary school might reflect a shift in strategy of letter processing, from single-letter processing to processing letters within syllables or words. Lastly, developmental trajectories of activation within the reading network varied depending on reading skills, indicating altered development of letter and speech-sound processing in children with dyslexia. Such differences were most pronounced when the task demanded matching audiovisual information, as in the case of the incongruency effect. In summary, our analyses of a fully longitudinal and a mixed longitudinal and cross-sectional sample of children with varying reading skills over five time points from kindergarten to fifth grade provide new insights into the trajectories of written and spoken letter processing and their integration in key regions of the reading network.

## Funding

This work was supported by the 10.13039/501100001711Swiss National Science Foundation (32003B_141201), the Hartmann Mueller Foundation (1912), the Olga Mayenﬁsch Foundation, Fondation Botnar (grant 6066), NCCR Evolving Language, 10.13039/501100001711Swiss National Science Foundation (agreement number 51NF40_180888) and the University Research Priority Program Adaptive Brain Circuits in Development and Learning (AdaBD) of the 10.13039/501100006447University of Zurich (project ChildBrainCircuits).

## CRediT authorship contribution statement

SD, IK, GP and SB designed research; SD, IK and GP performed research; SD, IK and SB analyzed data; SD, IK and SB wrote the paper; SB acquired funding and provided resources; All authors contributed to the editing of the manuscript and approved the submitted version.

## Declaration of Competing Interest

The authors declare that they have no known competing financial interests or personal relationships that could have appeared to influence the work reported in this paper.

## Data Availability

The data supporting the conclusions of this article will be made available by the authors upon reasonable request. Some restrictions may apply for data sharing due to the restricted consent of research participants. Scripts are available via Open Science Framework (OSF), https://doi.org/10.17605/OSF.IO/FWM6R.
