# Supplementary material for: Neurodevelopmental trajectories of letter and speech sound processing from preschool to the end of elementary school
Source: Dev Cogn Neurosci. 2023 May 12;61:101255. doi: 10.1016/j.dcn.2023.101255 (PMC10203735; doi:10.1016/j.dcn.2023.101255)
Supplement: Supplementary file 1 — Supplementary material [file mmc1.docx]

**Supplementary material**

**Neurodevelopmental trajectories of letter and speech sound processing from preschool to the end of elementary school**

Di Pietro, S. V.^a,b,c^, Karipidis, I. I.^a,b^, Pleisch, G.^a^, Brem, S.^a,b,c^

^a^Department of Child and Adolescent Psychiatry and Psychotherapy, University Hospital of Psychiatry Zurich, University of Zurich, Switzerland
^b^Neuroscience Center Zurich, University of Zurich and ETH Zurich, Switzerland
^c^URPP Adaptive Brain Circuits in Development and Learning (AdaBD), University of Zurich, Zurich, Switzerland

**5 Supplementary Methods**

**5.1 Motion correction and flagging**

For the fully longitudinal sample (n=16), at least one scan was repaired using the ArtRepair toolbox for 4 cases for T1 (m=4.23% of scans), 5 cases for T2 (m=1.17% of scans), 3 cases for T3 (m=1.59% of scans), 7 cases for T4 (m=6.05% of scans), and 2 cases for T5 (m=2.12% of scans). Flagging was performed in 4 cases for T1 (m=3.44% of scans), 1 case for T2 (1.59% of scans), 1 case in T3 (m=2.65% of scans), 5 cases for T4 (m=6.03% of scans), and no cases for T5.

For the mixed-longitudinal/cross-sectional sample, at least one scan was repaired for 15/35 cases for T1 (m=5.08% of scans), 19/44 cases for T2 (m=4.57% of scans), 13/43 cases for T3 (m=3.34% of scans), 17/38 cases for T4 (m=4.70% of scans), and 13/78 cases for T5 (m=1.83% of scans). Flagging was performed in 12 cases for T1 (m=4.76% of scans), 11 cases for T2 (m=5.58% of scans), 7 cases for T3 (m=3.33% of scans), 11 cases for T4 (m=4.62% of scans), and 2 cases for T5 (m=1.86% of scans).

Mean framewise displacement values (c.f. Supplementary Table 1) across all time points did not differ between the whole sample and the subsample without ADHD (*t*(461)=.117, *p*=.907), the whole sample and the subsample with ADHD (*t*(252)=-1.36, *p*=.175) or the subsamples without and with ADHD (*t*(239)=-1.39, *p*=.166).

| ***Supplementary Table 1*.** Framewise displacement per time point for the whole sample, the subsample without ADHD and the subsample with ADHD. | | | | |
| --- | --- | --- | --- | --- |
| **T1** | **T2** | **T3** | **T4** | **T5** |
| mean(SD), range | mean(SD), range | mean(SD), range | mean(SD), range | mean(SD), range |
| **Whole sample** | | | | |
| 0.31 (0.18),  0.10-0.85 | 0.33 (0.20),  0.12-0.94 | 0.24 (0.11),  0.10-0.54 | 0.33 (0.18),  0.11-0.91 | 0.21 (0.08), 0.08-0.53 |
| **Subsample without ADHD** | | | | |
| 0.30 (0.18),  0.10-0.85 | 0.34 (0.20),  0.12-0.94 | 0.24 (0.10),  0.10-0.54 | 0.32 (0.17),  0.11-0.91 | 0.20 (0.08),  0.08-0.53 |
| **Subsample with ADHD** | | | | |
| 0.43 (0.09),  0.34-0.52 | 0.24 (0.10),  0.13-0.38 | 0.28 (0.17),  0.10-0.50 | 0.47 (0.22),  0.22-0.75 | 0.29 (0.06),  0.21-0.40 |
| *Notes*. n=35 (T1), n=44 (T2), n=43 (T3), n=38 (T4), n=78 (T5) for the whole sample; n=33 (T1), n=41 (T2), n=40 (T3), n=35 (T4), n=73 (T5) for the subsample without ADHD; n=2 (T1), n=3 (T2), n=3 (T3), n=3 (T4), n=5 (T5) for the subsample with ADHD | | | | |

**5.2 Neurocognitive tests**

The rapid automatized naming (RAN) of the “Test zur Erfassung der phonologischen Bewusstheit und der Benennungsgeschwindigkeit” (TEPHOBE; Mayer (2011)) was performed at all time points for the subtests colors and objects and from T2 to T5 additionally for letters and digits. Phonological awareness was assessed with subtests of two standardized tests (see Supplementary Tables 2 and 3). We used the subtests rhyme and initial sound categorization from the TEPHOBE (Mayer, 2011). In the rhyme subtest, children are asked to select the two rhyming words out of four words presented auditorily. In the initial sound categorization subtest, they are asked to select two out of four words starting with the same speech sound. Both subtests contain seven items. Further, we used the subtests phoneme deletion and pseudoword segmentation from the Basiskompetenzen für Lese-Rechtschreibleistungen (BAKO; Stock et al. (2003)). In the phoneme deletion subtest, children are presented with words and pseudowords auditorily and have to repeat it without the initial speech sound. This subtest contains seven items. In the pseudoword segmentation subtest, children are asked to verbally segment the auditorily presented pseudowords. This subtest contains eight items. Finally, because most children at the end of kindergarten were able to name and/or partly write the (upper-case) letters of their given names, we also used an in-house word list, consisting of 20 highly frequent, short (2-5 letters) words (e.g. MAMA, PAPA etc.) written in capital letters at T1 to explore precursor decoding skills of children at kindergarten stage, when the children have not yet received formal reading instruction (reading instruction starts in grade 1, after kindergarten). We asked the children to try to read as many of the words as they could (no time restriction). 12/35 children were not able to read any of the simple words, 12/35 children were able to decode 1-3 simple words, 9/35 children were able to read or decode 4-10 simple words (most of the time the children spelled out the letters of the words rather than reading and understanding the words) and 2/35 children were able to read/decode more than 10 items.

| ***Supplementary Table 2*.** Descriptive statistics showing reading scores at each time point for the cross-sectional sample. | | | | | |
| --- | --- | --- | --- | --- | --- |
|  | **T1 (n=35)** | **T2 (n=44)** | **T3 (n=43)** | **T4 (n=38)** | **T5 (n=78)** |
|  | mean(SD), range | mean(SD), range | mean(SD), range | mean(SD), range | mean(SD), range |
| **Letter-knowledge^a^** | | | | | |
| Sounds | 14.91 (10.64), 1-47 | 44.52 (6.56), 21-52 | 47.84 (2.96), 41-52 | 49.37 (2.95), 41-52 | - |
| **Rapid automatized naming^e^** | | | | | |
| Letters | - | 1.04 (0.32), 0.36-1.79^c^ | 1.32 (0.37), 0.53-2.17 | 1.44 (0.39), 0.79-2.17 | 2.23 (0.49), 1.19-3.54 |
| Digits | - | 1.01 (0.26), 0.53-1.81^c^ | 1.13 (0.30), 0.56-1.79 | 1.34 (0.37), 0.68-2.17 | 2.08 (0.52), 0.98-3.55 |
| Objects | 0.59 (0.13), 0.29-0.89 | 0.69 (0.14), 0.35-1.04 | 0.75 (0.18), 0.40-1.09 | 0.87 (0.17), 0.47-1.28 | 1.14 (0.19), 0.77-1.61 |
| Colors | 0.56 (0.16), 0.27-0.89^b^ | 0.66 (0.25), 0.24-1.47 | 0.67 (0.23), 0.28-1.22 | 0.81 (0.24), 0.53-1.43 | 1.02 (0.24), 0.58-1.80 |
| **Phonological awareness** | | | | | |
| TEPHOBE |  |  |  |  |  |
| Rhyme | 5.06 (1.22), 2-7 | 5.05 (1.38), 2-7 | 5.30 (1.11), 3-7 | - | - |
| Initial sound categorization | 2.74 (1.86), 0-7 | 5.61 (1.81), 2-7 | 6.19 (1.08), 2-7 | - | - |
| BAKO |  |  |  |  |  |
| Phoneme deletion | 0.60 (1.52), 0-6 | 2.95 (2.12), 0-6^d^ | 3.21 (2.06), 0-7 | - | - |
| Pseudoword segmentation | 0.71 (1.32), 0-7 | 3.43 (1.91), 0-8 | 4.42 (1.73), 0-8 | - | - |
| **Decoding words**^f^ | | | | | |
| Word decoding | 3.11 (4.28),  0-19 |  |  |  |  |
| *Notes*. ^a^Sum of scores for items in lower and upper case (max score = 52); ^b^2 missing cases (n= 33); ^c^1 missing case (n=43); ^d^1 missing case (n=43); ^e^Measure: Number of items named per second; ^f^In-house word reading test, measure: number of words spelled out (maximum: 20 words all written in capital letters). PR = percentile score. Raw scores are: number of correct items (letter-knowledge, TEPHOBE, BAKO) and number of items per second (rapid automatized naming). | | | | | |

| ***Supplementary Table 3*.** Descriptive statistics showing reading scores at each time point for the longitudinal sample. | | | | | |
| --- | --- | --- | --- | --- | --- |
|  | **T1 (n=16)** | **T2 (n=16)** | **T3 (n=16)** | **T4 (n=16)** | **T5 (n=16)** |
|  | mean(SD), range | mean(SD), range | mean(SD), range | mean(SD), range | mean(SD), range |
| **Letter-knowledge^a^** | | | | | |
| Sounds | 20.06 (10.96), 2-47 | 45.06 (7.21),  23-52 | 48.69 (3.08),  42-52 | 50.25 (2.84),  41-52 | - |
| **Rapid automatized naming**^e^ | | | | | |
| Letters | - | 0.96 (0.27), 0.45-1.47 | 1.19 (0.37), 0.60-2.17 | 1.28 (0.38), 0.79-2.08 | 1.97 (0.45), 1.32-3.09 |
| Digits | - | 0.94 (0.27), 0.53-1.81 | 1.02 (0.26), 0.65-1.79 | 1.17 (0.30), 0.79-1.72 | 1.85 (0.48), 0.99-2.86 |
| Objects | 0.58 (0.12),  0.29-0.79 | 0.69 (0.10), 0.52-0.93 | 0.78 (0.15), 0.53-1.06 | 0.84 (0.16), 0.61-1.11 | 1.10 (0.18), 0.83-1.40 |
| Colors | 0.55 (0.16),  0.27-0.85 | 0.68 (0.26), 0.42-1.47 | 0.70 (0.22), 0.46-1.22 | 0.76 (0.23), 0.53-1.43 | 0.97 (0.21), 0.69-1.49 |
| **Phonological awareness** | | | | | |
| TEPHOBE |  |  |  |  |  |
| Rhyme | 4.88 (1.27), 2-6 | 5.13 (1.45), 2-7 | 5.63 (1.06), 3-7 | - | - |
| Initial sound categorization | 2.88 (1.83), 0-7 | 6.06 (1.64), 2-7 | 6.44 (0.61), 5-7 | - | - |
| BAKO |  |  |  |  |  |
| Phoneme deletion | 0.75 (1.82), 0-6 | 3.38 (2.06), 0-6 | 3.38 (1.80), 0-6 | - | - |
| Pseudoword segmentation | 0.06 (1.71), 0-7 | 3.48 (1.76), 1-6 | 3.75 (1.52),  0-6 | - | - |
| **Decoding words**^f^ | | | | | |
| Word decoding | 4.5 (5.15),  0-19 |  |  |  |  |
| *Notes*. ^a^Sum of scores for items in lower and upper case (max score = 52). ^b^2 missing cases (n= 33); ^c^1 missing case (n=43); ^d^1 missing case (n=43); ^e^Measure: Number of items named per second; ^f^In-house word reading test, measure: number of words spelled out (maximum: 20 words all written in capital letters). PR = percentile score. Raw scores are: number of correct items (letter-knowledge, TEPHOBE, BAKO) and number of items per second (rapid automatized naming). | | | | | |

**6 Supplementary results**

**6.1 Whole-brain analyses with covariate reading fluency**

| ***Supplementary Table 4.*** Whole brain analyses (n=16) with covariate reading fluency percentiles (log transformed) | | | | | | | |
| --- | --- | --- | --- | --- | --- | --- | --- |
| **Contrast** | **brain area** | **MNI coordinates** | | | **Cluster size** | **F/T-value** | **cluster-level p_FWEcorr_** |
|  |  | **x** | **y** | **z** |  |  |  |
| **Visual processing** | | | | | | | |
| **Main effect time point** | Lingual gyrus L | -20 | -81 | -9 | 97 | 12.50 | .006 |
|  | Fusiform gyrus R | 19 | -84 | -3 | 109 | 8.66 | .003 |
| **T2>T1** | SPL R | 19 | -69 | 42 | 158 | 3.98 | .004 |
| **T5>T1** | OCC L | -20 | -81 | -6 | 232 | 5.49 | <.001 |
|  | OCC R | 16 | -93 | 18 | 173 | 4.31 | .002 |
| **T5>T2** | Occipital pole L | -11 | -99 | 3 | 208 | 5.44 | .001 |
|  | OCC R | 16 | -87 | -3 | 88 | 4.04 | .046 |
| **T5>T3** | Fusiform gyrus L | -20 | -81 | -9 | 150 | 5.90 | .005 |
|  | OCC R | 34 | -78 | -6 | 182 | 4.61 | .002 |
| **T5>T4** | Fusiform gyrus L | -20 | -81 | -9 | 175 | 5.87 | .002 |
|  | Fusiform gyrus R | 28 | -78 | -9 | 301 | 5.47 | <.001 |
| **Auditory processing** | | | | | | | |
| **T1>T2** | Basal forebrain L | -2 | 3 | -9 | 87 | 4.98 | .040 |
|  | Precuneus R | 7 | -66 | 18 | 219 | 4.48 | <.001 |
| **T2>T5** | Heschl’s gyrus L | -38 | -30 | 15 | 153 | 4.65 | .004 |
| **Audiovisual processing** | | | | | | | |
| **Main effect time point** | OCC L | -11 | -96 | 9 | 565 | 16.02 | <.001 |
|  | OCC R | 34 | -78 | -9 | 614 | 15.00 | <.001 |
|  | STG L | -47 | -21 | 6 | 380 | 13.27 | <.001 |
| **T2>T1** | PT L | -53 | -24 | 6 | 573 | 5.51 | <.001 |
|  | STG R | 67 | -24 | 0 | 363 | 5.40 | <.001 |
| **T2>T5** | STG L | -47 | -21 | 6 | 251 | 5.45 | <.001 |
| **T3>T1** | PO L | -47 | -21 | 6 | 304 | 4.62 | <.001 |
| **T3>T5** | Heschl’s gyrus L | -44 | -21 | 6 | 140 | 5.68 | .009 |
| **T5>T1** | OCC bilateral | -11 | -96 | 9 | 1914 | 6.66 | <.001 |
|  | SPL L | -26 | -69 | 63 | 244 | 5.13 | <.001 |
|  | SPL R | 22 | -72 | 54 | 199 | 4.10 | .002 |
| **T5>T2** | OCC L | -11 | -96 | 9 | 438 | 6.64 | <.001 |
|  | OCC R | 13 | -93 | 18 | 337 | 5.41 | <.001 |
| **T5>T3** | OCC bilateral | 34 | -78 | -9 | 1070 | 6.16 | <.001 |
| **T5>T4** | SOG R | 31 | -78 | -6 | 883 | 6.44 | <.001 |
|  | Occipital pole L | -8 | -96 | 6 | 104 | 5.53 | .030 |
|  | OCC L | -20 | -81 | -9 | 552 | 5.34 | <.001 |
| ***Notes***. Cluster-defining threshold p=.001. Results are cluster-level FWE corrected p<.05. Abbreviations: OCC, occipital region; PO, parietal operculum; PT, planum temporale; SPL, superior parietal lobule; SOG, superior occipital gyrus; STG, superior temporal gyrus; L, left; R, right. | | | | | | | |

**6.2 LMM without ADHD-subsample**

The results of the subsample without ADHD differed in some aspects from the analyses with the whole sample: In the mixed longitudinal/cross-sectional sample, the main effect of time point for the congruent condition in the vOTC did not reach significance. The effect of reading fluency during congruent processing in the mSTG and the pSTG did not reach significance. In the longitudinal sample, the main effect of time point for the auditory condition in the pSTG (model with covariate) and for the congruent condition (model without covariate) in the IFG did not reach significance.

| ***Supplementary Table 5***. Summary of the ROI analyses without ADHD-subsample. | | | | | |
| --- | --- | --- | --- | --- | --- |
| **ROI** | **Condition** | **Effect** | **Longitudinal (no cov.);**  **rmANOVA** | **longitudinal (cov. RF);**  **rmANOVA** | **mixed longitudinal/cross-sectional (cov. RF); LMM** |
| vOTC | V | TP | **F(4,52)=2.57, p=.049** | n.s. (p=.578) | **F(4,144.19)=3.35, p=.012** |
|  |  | TP*RF | - | n.s. (p=.310) | F(4,144.70)=2.12, p=.081 |
|  |  | RF | - | n.s. (p=.366) | n.s. (p=.607) |
|  | A | TP | n.s. (p=.307) | n.s. (p=.340) | F(4,148.31)=2.05, p=.091 |
|  |  | TP*RF | - | n.s. (p=.536) | n.s. (p=.132) |
|  |  | RF | - | n.s. (p=.741) | n.s. (p=.917) |
|  | AV cong | TP | **F(4,52)=4.95, p=.002** | n.s. (p=.551) | F(4,126.59)=2.29, p=.063* |
|  |  | TP*RF | - | n.s. (p=.303) | n.s. (p=.606)* |
|  |  | RF | - | n.s. (p=.408) | n.s. (p=.435)* |
|  | Inco effect | TP | n.s. (p=.648) | F(2.5,30.3)=2.70, p=.072 | n.s. (p=.113)* |
|  |  | TP*RF | - | F(2.5,30.3)=2.56, p=.082 | **F(4,1169.11)=2.98, p=.021*** |
|  |  | RF | - | n.s. (p=.186) | n.s. (p=.439)* |
| mSTG | V | TP | n.s. (p=.366) | n.s. (p=.788) | n.s. (p=.986) |
|  |  | TP*RF | - | n.s. (p=.972) | n.s. (p=.379) |
|  |  | RF | - | n.s. (p=.368) | n.s. (p=.895) |
|  | A | TP | **F(4,52)=2.94, p=.029** | n.s. (p=.580) | n.s. (p=.647) |
|  |  | TP*RF | - | n.s. (p=.412) | n.s. (p=.178) |
|  |  | RF | - | n.s. (p=.257) | F(1,77.04)=2.89, p=.093 |
|  | AV cong | TP | **F(4,52)=3.48, p=.014** | n.s. (p=.555) | n.s. (p=.434) |
|  |  | TP*RF | - | n.s. (p=.807) | n.s. (p=.433) |
|  |  | RF | - | F(1,12)=3.38, p=.091 | n.s. (p=.128) |
|  | Inco effect | TP | n.s. (p=.908) | n.s. (p=.264) | n.s. (p=.309) |
|  |  | TP*RF | - | n.s. (p=.258) | n.s. (p=.103) |
|  |  | RF | - | n.s. (p=.281) | n.s. (p=.211) |
| pSTG | V | TP | n.s. (p=.269) | n.s. (p=.765) | n.s. (p=.287) |
|  |  | TP*RF | - | n.s. (p=.903) | n.s. (p=.399) |
|  |  | RF | - | n.s. (p=.440) | n.s. (p=.927) |
|  | A | TP | n.s. (p=.597) | n.s. (p=.762) | **F(4,133.65)=3.10, p=.018** |
|  |  | TP*RF | - | n.s. (p=.669) | **F(4,134.97)=3.24, p=.014** |
|  |  | RF | - | F(1,12)=4.39, p=.058 | n.s. (p=.409) |
|  | AV cong | TP | n.s. (p=.416) | n.s. (p=.749) | F(4,142.56)=2.07, p=.088 |
|  |  | TP*RF | - | n.s. (p=.493) | **F(4,143.77)=3.37, p=.011** |
|  |  | RF | - | n.s. (p=.356) | n.s. (p=.134) |
|  | Inco effect | TP | n.s. (p=.161) | n.s. (p=.665) | **F(4,176.92)=2.62, p=.037** |
|  |  | TP*RF | - | n.s. (p=.754) | **F(4,176.15)=2.91, p=.023** |
|  |  | RF | - | n.s. (p=.246) | n.s. (p=.951) |
| IFG | V | TP | n.s. (p=.503) | n.s. (p=.741) | n.s. (p=.534) |
|  |  | TP*RF | - | n.s. (p=.853) | n.s. (p=.417) |
|  |  | RF | - | n.s. (p=.474) | n.s. (p=.361) |
|  | A | TP | n.s. (p=.731) | n.s. (p=.315) | **F(4,161.08)=3.25, p=.014** |
|  |  | TP*RF | - | n.s. (p=.341) | **F(4,160.70)=2.64, p=.036** |
|  |  | RF | - | n.s. (p=.544) | n.s. (p=.305) |
|  | AV cong | TP | F(4,52)=2.10, p=.094 | n.s. (p=.538) | n.s. (p=.268) |
|  |  | TP*RF | - | n.s. (p=.800) | n.s. (p=.384) |
|  |  | RF | - | n.s. (p=.230) | **F(1,58.88)=9.40, p=.003** |
|  | Inco effect | TP | n.s. (p=.190) | **F(4,48)=2.65, p=.045** | **F(4,152.82)=2.84, p=.026** |
|  |  | TP*RF | - | **F(4,48)=2.59, p=.049** | F(4,153.27)=2.03, p=.093 |
|  |  | RF | - | n.s. (p=.540) | n.s. (p=.702) |
| *Notes*. List of main effects and interactions for the different models: Longitudinal without covariate (analyzed with an rmANOVA), longitudinal with covariate reading fluency (analyzed with an rmANOVA) and mixed longitudinal/cross-sectional (analyzed with an LMM). Significant effects and interactions of p<.05 are printed in bold, trends are printed in regular font. Asterisks mark models with non-normally distributed residuals that should be interpreted with caution. Abbreviations: rmANOVA, repeated measures analysis of variance; LMM; linear mixed model; ROI, region of interest; cov, covariate; TP, time point; RF, reading fluency; vOTC, visual occipitotemporal cortex; mSTG, middle superior temporal gyrus; pSTG, posterior superior temporal gyrus; IFG, inferior frontal gyrus; V, visual condition; A, auditory condition; AV cong, audiovisual congruent condition; inco effect, incongruency effect. | | | | | |

**6.3 Developmental trajectories in the LFA ROI**

An additional letter form area (LFA) ROI in the vOTC was defined based on previous studies suggesting a posterior activation of the vOTC during letter processing. For the LFA ROI, in the longitudinal sample without covariate reading fluency, there was a significant main effect of time point for the visual condition (*F*(4,60)=3.38, *p*=.015), showing a significant model fit for a cuartic function (*F*(1,15)=5.58, *p*=.032) and significantly stronger activation at T5 than T4 (*p_corr_*=.032). Additionally, there was a significant main effect of time point for the congruent condition (*F*(4,60)=3.59, *p*=.011), showing a linear trend (*F*(1,15)=7.93, *p*=.013) and cubic trend (*F*(1,15)=5.93, *p*=.028), with significantly stronger activation at T5 than T1 (*p_corr_*=.022).

In the mixed longitudinal/cross-sectional sample, LMMs showed a significant effect of time point for the visual condition (*F*(4,144.51)=3.20, *p*=.015), with significantly stronger activation at T5 than T1, T2, T3 and T4 (*p*s*_corr_*≤.006), and a significant main effect of time point for the auditory condition (*F*(4,170.44)=2.94, *p*=.022), with significantly stronger activation at T1 than T3 (*p_corr_*=.025) and than T4 (*p_corr_*=.002). The results are depicted in Supplementary Table 6 and Supplementary Figure 1.

| ***Supplementary Table 6***. Summary of the analyses in the LFA ROI. | | | | | |
| --- | --- | --- | --- | --- | --- |
| **ROI** | **Condition** | **Effect** | **Longitudinal (no cov.);**  **rmANOVA** | **longitudinal (cov. RF); rmANOVA** | **mixed longitudinal/cross-sectional (cov. RF); LMM** |
| LFA | V | TP | **F(4,60)=3.38, p=.015** | n.s. (p=.358) | **F(4,144.51)=3.20, p=.015** |
|  |  | TP*RF | - | n.s. (p=.577) | n.s. (p=.485) |
|  |  | RF | - | n.s. (p=.652) | n.s. (p=.581) |
|  | A | TP | F(4,60)=2.07, p=.096 | n.s. (p=.280) | **F(4,170.44)=2.94, p=.022** |
|  |  | TP*RF | - | n.s. (p=.393) | n.s. (p=.290) |
|  |  | RF | - | n.s. (p=.981) | n.s. (p=.366) |
|  | AV cong | TP | **F(4,60)=3.59, p=.011** | n.s. (p=.295) | F(4,131.14)=2.24, p=.068 |
|  |  | TP*RF | - | n.s. (p=.169) | n.s. (p=.314) |
|  |  | RF | - | n.s. (p=.207) | n.s. (p=.414) |
|  | Inco effect | TP | F(4,60)=2.20, p=.079 | F(4,56)=2.36, p=.065 | n.s. (p=.308) |
|  |  | TP*RF | - | n.s. (p=.321) | n.s. (p=.357) |
|  |  | RF | - | F(1,14)=3.91, p=.068 | n.s. (p=.153) |
| *Notes.* List of main effects and interactions for the different models: Longitudinal without covariate (analyzed with an rmANOVA), longitudinal with covariate reading fluency (analyzed with an rmANOVA) and mixed longitudinal/cross-sectional (analyzed with an LMM). Significant effects and interactions of p<.05 are printed in bold, trends are printed in regular font. Abbreviations: rmANOVA, repeated measures analysis of variance; LMM; linear mixed model; ROI, region of interest; cov, covariate; TP, time point; RF, reading fluency; LFA, letter form area; V, visual condition; A, auditory condition; AV cong, audiovisual congruent condition; inco effect, incongruency effect. | | | | | |


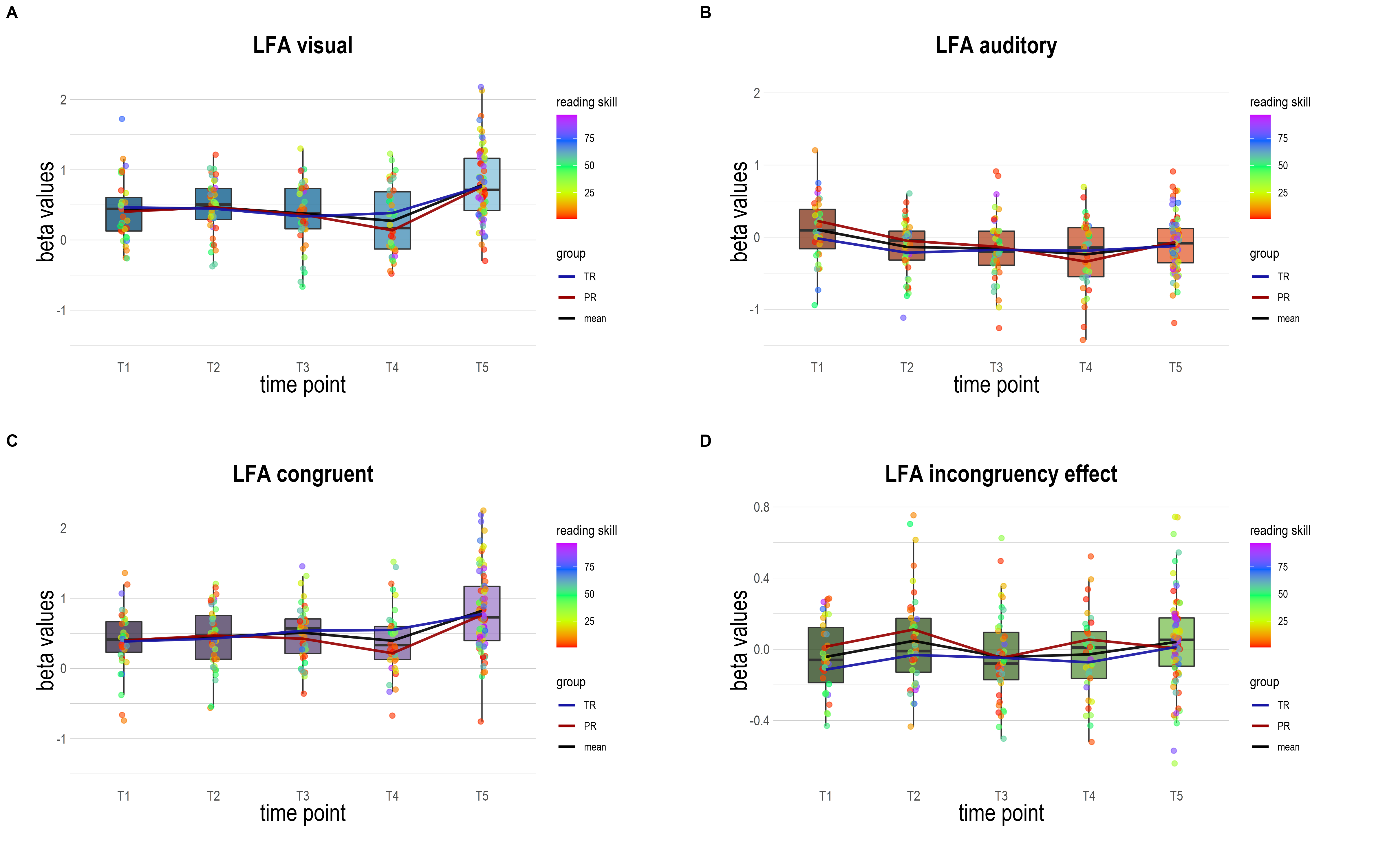


**Supplementary Figure 1**. Results of the ROI analyses for the pooled longitudinal and cross-sectional sample within the LFA. Depicted are the beta values extracted A) during visual letter processing B) during auditory speech sound processing, C) during congruent audiovisual processing, and D) for the audiovisual incongruency effect per time point. T1: kindergarten; T2: Middle of 1^st^ grade; T3: End of 1^st^ grade; T4: Middle of 2^nd^ grade; T5: Middle of 5^th^ grade. Developmental trajectories of activation are shown in black for the whole sample and separately for children considered as typical readers (blue line) or poor readers (red line), colored dots represent single subjects, and the colors reflect individual reading skill percentiles as indicated by the color bar. Abbreviations: ROI, region of interest; LFA, letter form area; TR, typical readers; PR, poor readers.

**Supplementary References**

Mayer, A. (2011). *Test zur Erfassung der phonologischen Bewusstheit und der Benennungsgeschwindigkeit (TEPHOBE)*. Reinhardt.

Stock, C., Marx, P., & Schneider, W. (2003). *Basiskompetenzen für Lese-Rechtschreibleistungen: BAKO 1-4; ein Test zur Erfassung der phonologischen Bewusstheit vom ersten bis vierten Grundschuljahr; Manual*. Beltz Test.
